# What affects natural killer cell activity: a cross-sectional study

**DOI:** 10.3389/fimmu.2026.1751240

**Published:** 2026-04-29

**Authors:** Junhyung Moon, Hyoju Oh, Yechan Yu, Eunkyung Suh, A-Ra Cho, Moon Jong Kim, Soo Hyun Lee, Jin Hun Park, Baek Hwan Cho, Yun-Kyong Lee

**Affiliations:** 1 Department of Software Convergence, School of Healthcare Convergence, CHA University, Pocheon, Republic of Korea; 2 Department of Biomedical Informatics, CHA University School of Medicine, CHA University, Seongnam, Republic of Korea; 3 Chaum Life Center, Department of Family Medicine, CHA University, Seoul, Republic of Korea; 4 Department of AI Healthcare Convergence, CHA University, Pocheon, Republic of Korea; 5 Department of Family Medicine, Gangnam Severance Hospital, Yonsei University College of Medicine, Seoul, Republic of Korea; 6 Department of Family Medicine, CHA Bundang Medical Center, CHA University School of Medicine, Seongnam, Republic of Korea; 7 Department of Family Medicine, Chaum Medical Checkup Center Samseongdong Branch, CHA University, Seoul, Republic of Korea

**Keywords:** artificial intelligence, health-screening data, immunity, natural killer cell activity, statistical analysis

## Abstract

**Introduction:**

Natural killer (NK) cells are key effectors of innate immunity, mediating rapid defense against infections and malignancies while maintaining tissue homeostasis. Despite its clinical relevance, few large-scale studies have comprehensively analyzed the diverse factors influencing NK cell activity (NKA).

**Methods:**

This study analyzed factors associated with NKA in 11,007 health-screening records integrating clinical, hematologic, metabolic, inflammatory, lifestyle, and self-reported symptom data.

**Results:**

46 check-up variables and 14 questionnaire items were significantly associated with NKA, including inflammatory markers such as neutrophil-to-lymphocyte ratio (NLR), platelet-to-lymphocyte ratio (PLR), platelet count, and monocyte count; metabolic indices including albumin, total protein, alkaline phosphatase (ALP), low-density lipoprotein (LDL)-cholesterol, calcium, and phosphorus; tumor markers such as carcinoembryonic antigen (CEA); lifestyle factors including smoking, exercise, and sleep quality; and subjective symptoms such as fatigue, dizziness, nocturia, myalgia, and heat intolerance. Artificial intelligence models captured nonlinear interactions among heterogeneous variables, achieving the highest area under the receiver operating characteristic curve of 0.716 ± 0.014 for identifying low-NKA individuals. SHAP analysis identified neutrophil count, PLR, and platelet count as top predictors.

**Discussion:**

Lower NKA was associated with elevated inflammatory markers and altered metabolic profiles, highlighting the interplay between systemic inflammation, metabolic dysregulation, and innate immune function. This study provides the first integrated, population-scale mapping of NKA association from routine health checkup data, demonstrating the feasibility of AI-driven immune profiling.

## 1 Introduction

Natural Killer (NK) cells represent a vital arm of the innate immune system, contributing substantially to the immediate defense against viral infections and malignancies (1). The significance of NK cells lies in their role as early responders, providing rapid and nonspecific immunity against emerging threats prior to the engagement of the adaptive immune system (2). This specialized subset of lymphocytes is adept at discerning healthy from aberrant cells through a finely tuned interplay of activating and inhibitory receptors on their surface (3). When confronted with cells expressing viral or neoplastic markers, the balance shifts toward activation, culminating in the release of potent lytic molecules like perforin and granzymes, and cytokines such as interferon-gamma (IFN-γ), orchestrating the targeted destruction of the identified anomalies (4). Beyond these classical roles, NK cells also participate in the clearance of senescent or dysfunctional cells, thereby mitigating chronic inflammation and maintaining tissue homeostasis—a process increasingly recognized as critical in healthy aging. Adequate NK cell activity (NKA) contributes to effective immune surveillance, preventing the progression of infections and the development of cancer by swiftly targeting and destroying aberrant cells, thereby upholding overall health and immune system functionality (5).

Several studies revealed that NKA is intricately influenced by diverse factors, such as age-related declines (6), chronic stress with high cortisol level (7), nutritional status (8), infections (9), hypertension (10), and even body composition (11). While these findings underscore the multifaceted correlates of NKA, most prior investigations have been limited by modest sample sizes or narrow clinical settings, often focusing on a restricted set of variables. A more systematic and large-scale evaluation is therefore essential to delineate the breadth of factors associated with NKA, as such knowledge is fundamental to understanding its variability in populations and to positioning NKA as one of the useful indicators for assessing immune health. Despite its clinical relevance, no large-scale study has comprehensively analyzed these associations. Public datasets containing validated NKA measurements are unavailable, and large private datasets are rare due to the cost and logistical burden of immune testing.

In this study, a substantial private dataset encompassing over 16,000 health-screening cases integrating clinical, biochemical, hematologic, and lifestyle data was secured. The study aimed to systematically identify the key factors associated with NKA and to further investigate the feasibility of an artificial intelligence (AI)-driven, multimodal model capable of predicting immune activity in real-world populations. We used AI because it can capture weak signals across multiple domains and possible nonlinear interactions that conventional univariate analyses may miss. Some of the known applications of AI on immune study are immunotherapy (12), precision oncology (13–15), and tumor pathology (16). Collectively, this work delivers the first integrated, population-scale map of correlates of NKA from routine health checkup data.

## 2 Materials and methods

### 2.1 Study design and participants

A retrospective observational study was conducted using data from a single medical facility, Chaum Life Center in the Republic of Korea. Adults aged ≥18 years who underwent health examination that included NKA measurement were enrolled between January 2016 and May 2024. Height and weight were measured while participants were standing without footwear and were recorded to the nearest decimal place in centimeters and kilograms, respectively. Waist circumference was measured according to the NHANES protocol (17). Body mass index (BMI) was calculated as weight in kilograms divided by height in meters squared. Blood pressure (BP) was measured using an automated sphygmomanometer after a 10-minute seated rest. Resting heart rate (RHR) was measured by palpating the right radial artery for 60 seconds. Morning venous blood samples were collected from the antecubital vein after an overnight fast of at least eight hours. Hematologic and biochemical analyses, including complete blood count and serum chemistry parameters, were performed using standardized automated analyzers within 24 hours of sample collection. NKA was assessed using a whole-blood assay (NK Vue^®^ Kit; NKMAX Co., Ltd., Seongnam, Republic of Korea) by measuring interferon-gamma (IFN-γ) secretion from activated NK cells. A 1 mL blood sample was incubated with a proprietary cytokine-containing NK Vue^®^ stimulation tube at 37 °C for 20–24 hours according to the manufacturer’s protocol. Although IFN-γ can be secreted by various immune cells, the NK Vue^®^ kit is designed to measure IFN-γ released predominantly from NK cells following cytokine stimulation (18–22). After incubation, plasma was separated and IFN-γ levels were quantified (pg/mL) using enzyme-linked immunosorbent assay (ELISA) provided as part of the NK Vue^®^ system. To ensure reliability, all assays were performed in duplicate, with standard curves and quality control procedures strictly followed. The study protocol was approved by the Institutional Review Board of CHA Bundang Medical Center (CHAMC 2022-09-045-001) and was conducted in accordance with the principles of the Declaration of Helsinki.

### 2.2 Data processing and statistical analysis

Each record was managed by a pseudo-identifier. The dataset comprised 100 health-examination variables, including age, sex, and NKA, and 31 questionnaire items capturing patients’ past and current status. From the 100 variables, we derived five additional features: muscle percentage (100 x muscle mass/weight), fat percentage (100 x fat mass/weight), NLR (neutrophil count/lymphocyte count), PLR (PLT/lymphocyte count), and WHR (waist circumference/height).

For both statistical analyses and AI model development, described below, we selected only each patient’s first examination to avoid potential bias from interventions after the initial results. In addition, any record with ≥30% missing data across variables was excluded to ensure adequate data completeness for statistical analyses and AI model development, minimizing the potential bias introduced by excessive imputation. In this work, we aimed to comprehensively examine the correlation between routine health checkup data and binary NKA status, categorized as high and low. A binary classification threshold of 250 was adopted based on prior studies (23, 24), defining the Low NKA Group (<250) and Non-low NKA Group (≥250). Additionally, to evaluate the robustness of our findings, statistical analyses and machine learning experiments in our study were also conducted using alternative thresholds of 100 and 500. These results are provided as sensitivity analyses in the **Supplementary Materials**.

As an exploratory step to identify potential candidate variables, we evaluated the associations between each variable and the binary NKA status using univariable Welch’s t-tests for numerical variables and Chi-squared tests for categorical variables and questionnaire responses. For statistically significant numerical variables (p-value<0.05), we examined each variable’s distribution according to the binary NKA status. For the statistically significant categorical variables and questionnaire responses, we investigated the distribution of NKA values across categories. To address the potential inflation of false-positive findings due to multiple comparisons, we calculated the false discovery rate (FDR)-adjusted p-values (q-values) for these univariable screenings.

Subsequently, to identify variables independently associated with binary NKA status, we additionally performed multivariable logistic regression analysis. All variables were grouped into seven clinically coherent categories (i.e., vital, body composition, hematologic indicators, metabolic and biochemical indicators, immunological and inflammatory markers, tumor markers, and urine analysis). For each category, a separate logistic regression model was constructed. In every model, eight covariates considered to be fundamental demographic and lifestyle-related confounders were included: age, sex, BMI, drinking status, smoking status, and self-reported use of antihypertensive, antidiabetic, and lipid-lowering medications. Within each category-specific model, the covariates were entered together with the domain-specific variables identified in the univariable screening. This framework allowed us to estimate the adjusted associations between each domain variable and NKA status while controlling for the same set of covariates across all models. Regression results were summarized using p-values, odds ratios (ORs), and 95% confidence intervals.

### 2.3 AI-based NKA status classification

We further investigated the feasibility of AI-based classification for binary NKA status using the statistically significant variables and questionnaire items that showed statistically significant associations with NKA status in the univariable analyses described above. Missing values were imputed with the median (continuous variables), the mode (categorical variables), and 0 for questionnaire items (0: absence of the condition, such as a disease, a symptom, or a past experience). Feature vectors were then constructed and z-score normalization was applied to continuous features. Given the novelty of utilizing routine health examination and questionnaire data for NKA binary classification, and the absence of an established standard algorithm for this specific task, it was essential to explore a wide range of fundamental models to verify general feasibility and establish a robust baseline performance. Therefore, we employed four machine learning models (Logistic Regression, Random Forest, XGBoost, CatBoost) and six deep learning models (Fully Connected Neural Network (FCNN, multilayer perceptron), TabNet (25), FT-Transformer (26), TabPFN (27), ExcelFormer (28), TabM (29)). These models were implemented in Python 3.11.4 using publicly available source codes from the authors’ GitHub repositories and distributed packages. Input data consisted of normalized feature vectors, and binary NKA labels served as ground truth. Model training used 5-fold subject-wise cross-validation with age-balanced folds (stratified by age) to mitigate potential age-related effects. Although the correlation between age and immune levels has not been conclusively demonstrated, we accounted for potential aging effects, given the general decline in physical functions associated with age.

The sklearn, xgboost, and catboost packages were used for Logistic Regression, Random Forest, XGBoost, and CatBoost models. For logistic regression, we fixed the solver to lbfgs with L2 regularization. For random forest and XGBoost, we tuned the number and depth of trees (and learning rate for XGBoost); for CatBoost, we tuned the number of iterations, maximum depth, and learning rate. We used the python packages and the source codes from the authors’ Github repositories to build six deep learning models. The FCNN (implemented with torch) stacked BatchNorm1d, Linear, ReLU, and Dropout layers; we tuned the number of layers/hidden units, epochs, and batch size. For TabNet, we adjusted the dimension of decision prediction layers, the dimension of the attentive transformer, the number of decision steps, the number of epochs, batch size, and learning rate. For FT-Transformer and ExcelFormer, we varied embedding dimension, the number of Transformer encoder layers, the number of attention heads, learning rate, and batch size. For TabPFN, we tuned the number of ensemble members. For TabM, we adjusted the number of ensemble blocks and the number of fully-connected layers.

Given the class imbalance in our dataset (Low NKA Group (class 1):Non-low NKA Group (class 0) ≈ 2.2:7.8), we applied cost-sensitive learning to penalize errors in the minority class more heavily. The binary cross-entropy loss was adjusted to assign a higher penalty for misclassification of minority-class instances (class 1).

To assess model performance, we computed area under receiver operating characteristic (AUROC), area under the precision recall curve (PRAUC), accuracy, precision, recall, specificity, and F1 score. When calculating these performance metrics, we adopted Youden’s index (30) to find the optimal threshold. Feature importance was further analyzed using the SHapley Additive exPlanations (SHAP) (31).

## 3 Results

### 3.1 Data characteristics

Our dataset comprised 16,322 records containing one or more routine health checkups per individual. Selecting only the initial examination for each individual yielded 11,254 records. After excluding two records from pregnant women, 11,252 records remained. Finally, we constructed the dataset of 11,007 records for statistical and AI-based analyses by excluding records for which missing rate across the entire 105 checkup variables was equal to or greater than 30%. Of the 11,007 patients included, the unadjusted descriptive statistics for demographic characteristics, lifestyles, clinical histories, and significant checkup variables are summarized in **Table 1**. The mean age was 48.0 ± 11.8 years, and females are 6,021, while males are 4,986. The study population was predominantly of Asian ethnicity (≈99%). Low NKA Group included 2,452 records (22.3%), and Non-low NKA Group included 8,555 records (77.7%), as shown in **Table 1**. The unadjusted descriptive statistics for the entire dataset, as well as for the binary NKA groups across all three thresholds (100, 250, and 500), are comprehensively detailed in **Supplementary Table 1**.

**Table 1:** Clinical characteristics of the study population (unadjusted descriptive statistics).

Variables	Total	Low NKA group (NKA<250)	Non-low NKA group (NKA≥250)
Participants, n	11,007	2,452	8,555
Age, years	47.98 ± 11.76	48.89 ± 11.74	47.72 ± 11.76
Male sex, n (%)	4,986 (45.3)	1,051 (42.9)	3,935 (46.0)
BMI, kg/m²	23.33 ± 3.76	23.25 ± 3.98	23.35 ± 3.70
SBP, mmHg	119.01 ± 13.59	119.82 ± 13.92	118.78 ± 13.49
DBP, mmHg	77.30 ± 10.96	77.95 ± 11.13	77.11 ± 10.91
RHR, bpm	74.39 ± 11.57	76.57 ± 12.32	73.77 ± 11.26
WBC, ×10³/µL	5.49 ± 1.59	6.05 ± 1.97	5.32 ± 1.42
Neutrophil, ×10³/µL	56.41 ± 9.27	61.08 ± 9.97	55.07 ± 8.60
Lymphocyte, ×10³/µL	33.45 ± 8.28	29.58 ± 8.56	34.57 ± 7.85
Monocyte, ×10³/µL	7.09 ± 1.90	6.61 ± 1.94	7.23 ± 1.86
Basophil, 10³/µL	0.56 ± 0.34	0.54 ± 0.34	0.56 ± 0.34
Platelet, ×10³/µL	253.44 ± 57.41	269.98 ± 64.39	248.70 ± 54.33
NLR	1.91 ± 1.07	2.45 ± 1.59	1.75 ± 0.80
PLR	8.23 ± 3.67	10.25 ± 5.24	7.65 ± 2.83
Total Cholesterol, mg/dL	204.14 ± 40.17	208.80 ± 42.03	202.81 ± 39.52
ALP, U/L	179.01 ± 57.56	187.39 ± 68.14	176.61 ± 53.91
Calcium, mg/dL	9.19 ± 0.40	9.23 ± 0.42	9.18 ± 0.40
Phosphorus, mg/dL	3.60 ± 0.47	3.63 ± 0.51	3.59 ± 0.46
CEA, ng/mL	1.68 ± 1.82	1.86 ± 3.24	1.63 ± 1.11
Alcohol, n (%)	7,584 (68.9)	1,636 (66.7)	5,948 (69.5)
Smoking, n (%)	1,899 (17.3)	457 (18.6)	1,442 (16.9)
Regular exercise, n (%)	7,201 (65.4)	1,551 (63.3)	5,650 (66.0)
Hypertension, n (%)	1,583 (14.4)	358 (14.6)	1,225 (14.3)
Diabetes mellitus, n (%)	599 (5.4)	156 (6.4)	443 (5.2)
Dyslipidemia, n (%)	1,551 (14.1)	367 (15.0)	1,184 (13.8)

Data are presented as mean± SD or number (%).

NKA, natural killer cell activity; BMI, body mass index; SBP, systolic blood pressure; DBP, diastolic blood pressure; RHR, Resting heart rate; WBC, white blood cell; NLR, neutrophil-to-lymphocyte ratio; PLR, platelet-to-lymphocyte ratio; ALP, alkaline phosphatase; CEA, carcinoembryonic antigen.

### 3.2 Statistically significant factors to NKA binary status

**Figure 1:**
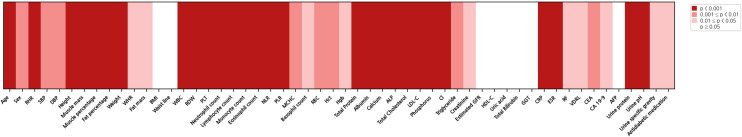
Heatmap illustrating the statistical significance of 54 health examination variables for NKA binary classification (threshold: 250). The cells are colored white (non-significant) or in three varying intensities of red based on their respective p-values.

**Figure 1** shows a heatmap illustrating the statistical significance (p<0.05) of health examination variables in the binary NKA classification scenario (threshold: 250) in the univariable analyses. We organized all variables into nine categories: demographics, vital, body composition, hematologic indicators, metabolic and biochemical indicators, immunological and inflammatory markers, tumor markers, urine analysis, and medication. Among these 54 variables, 46 were statistically significant: demographics (age, sex), vital (RHR, SBP, DBP), body composition (fat percentage, height, muscle mass, muscle percentage, weight, WHR, fat mass), hematologic indicators (basophil count, eosinophil count, Hct, Hgb, lymphocyte count, MCHC, monocyte count, neutrophil count, NLR, PLR, PLT, RBC, RDW, WBC), metabolic and biochemical indicators (albumin, ALP, calcium, Cl, LDL-c, phosphorus, total cholesterol, triglyceride), immunological and inflammatory markers (CRP, ESR, RF, VDRL), tumor markers (CA 19-9, CEA), urine analysis (urine PH, urine protein, urine specific gravity), and medication (antidiabetic medication). The corresponding univariable screening results for the alternative thresholds (100 and 500) are presented in **Supplementary Figure 1**.

**Figure 2:**
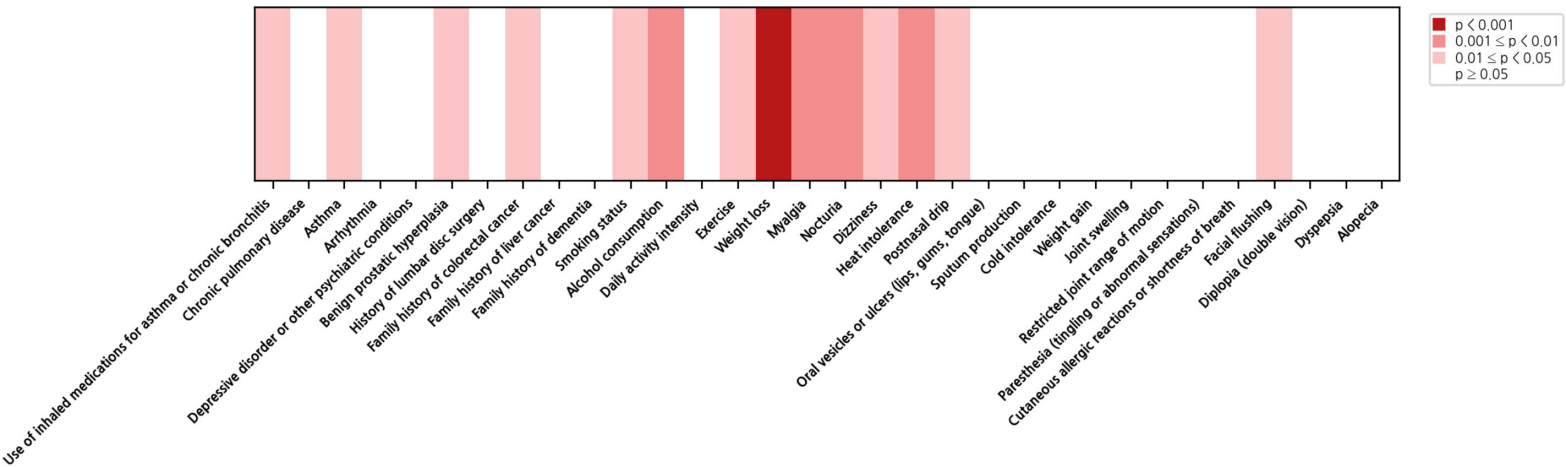
Heatmap illustrating the statistical significance of 32 questionnaire items for NKA binary classification (threshold: 250). The cells are colored white (non-significant) or in three varying intensities of red based on their respective p-values.

**Figure 2** shows a heatmap illustrating the statistical significance (p<0.05) of questionnaire items in the binary NKA classification scenario (threshold: 250) in the univariable analyses. All items were grouped into four categories: medical history, lifestyle, current symptom, and demographics. Among these 32 variables, 14 were statistically significant across all scenarios: medical history (asthma, benign prostatic hyperplasia, family history of colorectal cancer, use of inhaled medications for asthma or chronic bronchitis), lifestyle (alcohol consumption, exercise, smoking status), and current symptom (dizziness, facial flushing, heat intolerance, myalgia, nocturia, postnasal drip, weight loss). The heatmap results for the alternative thresholds (100 and 500) are illustrated in **Supplementary Figure 2**.

For the statistically significant numerical variables (p<0.05) identified in the univariable screening, we visualized the distribution of each variable’s values according to the binary NKA classes (low NKA vs. non-low NKA) using interquartile range (IQR) plots. These IQR plots for the thresholds of 100, 250, and 500 are presented in **Supplementary Figures 3**–**5**, respectively. Additionally, for the questionnaire items with binary options, the distributions of continuous NKA values comparing the ‘Yes’ and ‘No’ response groups were visualized using IQR plots, as shown in **Supplementary Figure 6**. Similarly, for items with multiple response options, the NKA value distributions across the respective categories are illustrated in **Supplementary Figure 7**. In addition, the detailed p-values and q-values for health examination variables and questionnaire items for thresholds of 100, 250, and 500 are provided in **Supplementary Tables 2**–**4**, respectively.

**Table 2** presents the results of the multivariate logistic regression for the NKA binary status. The multivariable logistic regression revealed various independent predictors of low NKA status: vital sign (particularly RHR), adiposity-related body-composition indicators (WHR), hematologic markers (RDW, PLT, RBC, Basophil count, NLR, WBC), metabolic and biochemical indicators (ALP, CI, total protein, creatinine, triglyceride, calcium), inflammatory markers (ESR, CRP), tumor marker (CEA), urine findings (urine protein and urine pH), medical history (family history of colorectal cancer, benign prostatic hyperplasia), lifestyle (exercise (2~4 times per week), exercise (4+ times per week)), and current symptom (weight loss, myalgia). The corresponding multivariable logistic regression results for the alternative thresholds of 100 and 500 are detailed in **Supplementary Tables 5**, **6**, respectively.

**Table 2:** Multivariate logistic regression results for binary NKA status using a threshold of 250 pg/mL.

Category	Variable/Item	P-value	OR	CI low	CI high
Vital	RHR	0.000	1.021	1.017	1.025
	SBP	0.060	1.005	1.000	1.011
	DBP	0.945	1.000	0.993	1.007
Body composition	WHR	0.005	18.534	2.394	143.499
	Fat mass	0.460	1.269	0.675	2.384
	Fat percentage	0.620	0.906	0.614	1.338
	Weight	0.637	0.859	0.457	1.613
	Height	0.647	1.008	0.974	1.043
	Muscle mass	0.798	1.091	0.561	2.120
	Muscle percentage	0.874	0.967	0.641	1.458
Hematologic indicators	RDW	0.000	1.206	1.132	1.285
	PLT	0.000	1.007	1.005	1.010
	RBC	0.000	0.594	0.460	0.768
	Basophil count	0.005	1.292	1.082	1.542
	NLR	0.011	1.288	1.059	1.566
	WBC	0.022	1.045	1.006	1.086
	Monocyte count	0.218	0.928	0.823	1.045
	PLR	0.350	0.969	0.907	1.035
	MCHC	0.432	1.158	0.803	1.668
	Neutrophil count	0.560	1.035	0.922	1.162
	Hct	0.596	1.082	0.808	1.451
	Lymphocyte count	0.802	0.985	0.877	1.107
	Eosinophil count	0.956	1.003	0.890	1.131
	Hgb	0.994	0.996	0.410	2.420
Metabolic & biochemical indicators	ALP	0.000	1.002	1.001	1.003
	Cl	0.000	1.036	1.017	1.055
	Total Protein	0.001	1.278	1.100	1.486
	Creatinine	0.014	0.659	0.472	0.920
	Triglyceride	0.026	1.001	1.000	1.001
	Calcium	0.035	1.172	1.011	1.358
	LDL-C	0.136	1.002	0.999	1.006
	Albumin	0.502	0.919	0.719	1.175
	Phosphorus	0.766	0.984	0.882	1.096
	Total Cholesterol	0.887	1.000	0.997	1.003
Immunological & inflammatory markers	ESR	0.000	1.022	1.016	1.027
	CRP	0.014	1.209	1.040	1.407
	RF	0.068	1.004	1.000	1.007
	VDRL	0.164	1.633	0.818	3.260
Tumor markers	CEA	0.000	1.116	1.072	1.162
	CA 19-9	0.059	1.004	1.000	1.009
Urine analysis	Urine protein	0.000	1.693	1.458	1.966
	Urine pH	0.000	0.901	0.853	0.952
	Urine specific gravity	0.410	15.621	0.023	10781.432
Medical history	Family history of colorectal cancer	0.029	1.214	1.021	1.445
	Benign prostatic hyperplasia	0.035	1.286	1.017	1.625
	Asthma	0.143	1.309	0.913	1.877
	Use of inhaled medications for asthma or chronic bronchitis	0.263	1.360	0.793	2.331
Lifestyle	Exercise (2~4/week)	0.002	0.84	0.751	0.938
	Exercise (4+/week)	0.018	0.816	0.69	0.965
	Exercise (2~4/month)	0.487	0.954	0.835	1.09
Current symptom	Weight loss	0.000	1.562	1.233	1.979
	Myalgia	0.019	1.176	1.027	1.346
	Postnasal drip	0.107	1.175	0.966	1.428
	Heat intolerance	0.113	1.143	0.969	1.349
	Nocturia	0.288	1.085	0.933	1.262
	Facial flushing	0.467	1.069	0.893	1.279
	Dizziness	0.659	1.033	0.894	1.193

*p*-value, OR, CI low, and CI high were calculated using the multivariate logistic regression where covariates were age, sex, BMI, drinking status, smoking status, and self-reported use of antihypertensive, antidiabetic, and lipid-lowering medications.

NKA, natural killer cell activity; OR, odds ratio; CI, confidence interval; RHR, Resting heart rate; SBP, systolic blood pressure; DBP, diastolic blood pressure; WHR, waist-to-height ratio; RDW, red cell distribution width; PLT, platelet count; RBC, red blood cell count; NLR, neutrophil-to-lymphocyte ratio; WBC, white blood cell; PLR, platelet-to-lymphocyte ratio; MCHC, mean corpuscular hemoglobin concentration; Hct, hematocrit; Hgb, hemoglobin; ALP, alkaline phosphatase; Cl, chloride; LDL-C, low-density lipoprotein cholesterol; ESR, erythrocyte sedimentation rate; CRP, C-reactive protein; RF, rheumatoid factor; VDRL, Venereal Disease Research Laboratory test; CEA, carcinoembryonic antigen; CA 19-9, carbohydrate antigen 19-9; BMI, body mass index.

Variables and questionnaire items are arranged in ascending order of p-values within each category.

### 3.3 AI-based classification results

**Figure 3:**
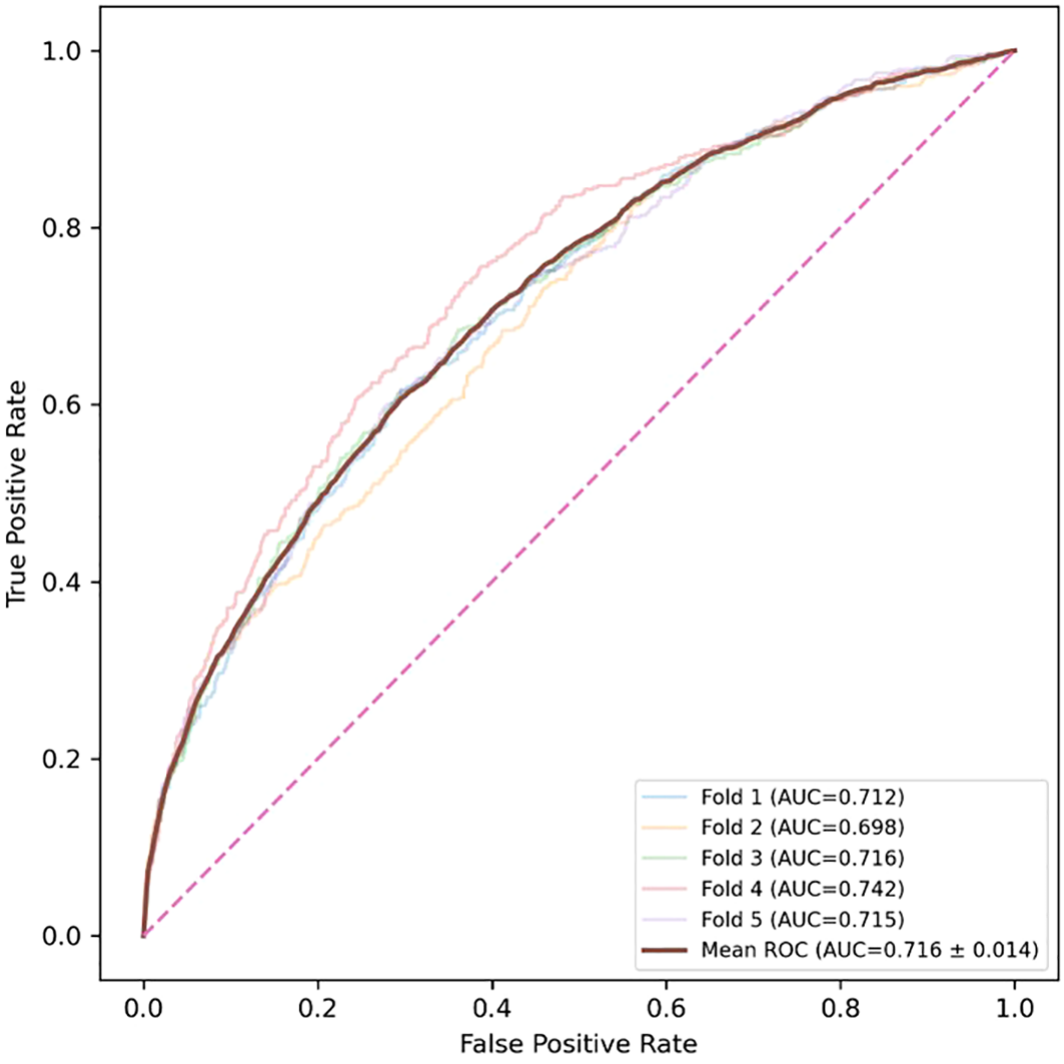
ROC curves for the TabPFN model (best AUROC 0.716 ± 0.014) based on 5-fold cross-validation.

Using the statistically significant variables and questionnaire items, we evaluated the feasibility of AI-based binary classification of NKA. The univariable analyses identified 60 significant variables and questionnaire items. These features formed the input vectors for the AI models, and the binary NKA classes served as labels. **Supplementary Table 7** presents the age and sex distributions across our subject-wise 5-fold cross-validation datasets. As presented in **Table 3**, TabPFN achieved the highest AUROC (0.716 (± 0.014)) with recall and specificity of 0.676 and 0.645, respectively. The corresponding ROC curve for the TabPFN model is illustrated in **Figure 3**. Additionally, splitting the dataset according to sex did not introduce any prominent differences in NKA binary classification results. **Supplementary Table 8** presents the binary classification results for the alternative thresholds of 100 and 500.

**Table 3:** NKA binary classification results for nine AI models using a threshold of 250 pg/mL.

AI model	AUROC	PRAUC	Accuracy	Precision	Recall	Specificity	F1 score
Linear Regression	0.713 ( ± 0.012)	0.452	0.665	0.359	0.643	0.671	0.461
Random Forest	0.703 ( ± 0.013)	0.443	0.664	0.358	0.624	0.677	0.452
XGBoost	0.704 ( ± 0.011)	0.438	0.656	0.353	0.644	0.659	0.455
CatBoost	0.713 ( ± 0.013)	0.456	0.675	0.367	0.628	0.689	0.462
FCNN	0.712 ( ± 0.014)	0.453	0.652	0.352	0.669	0.647	0.461
TabNet	0.708 ( ± 0.012)	0.441	0.656	0.353	0.655	0.657	0.459
FT-Transformer	0.709 ( ± 0.014)	0.446	0.656	0.354	0.658	0.656	0.460
TabPFN	**0.716 ( ± 0.014**)	0.458	0.652	0.356	0.676	0.645	0.464
ExcelFormer	0.712 ( ± 0.016)	0.453	0.659	0.358	0.658	0.660	0.463
TabM	0.707 ( ± 0.018)	0.446	0.654	0.353	0.657	0.653	0.459

Bold means the best AUROC value.

**Figure 4:**
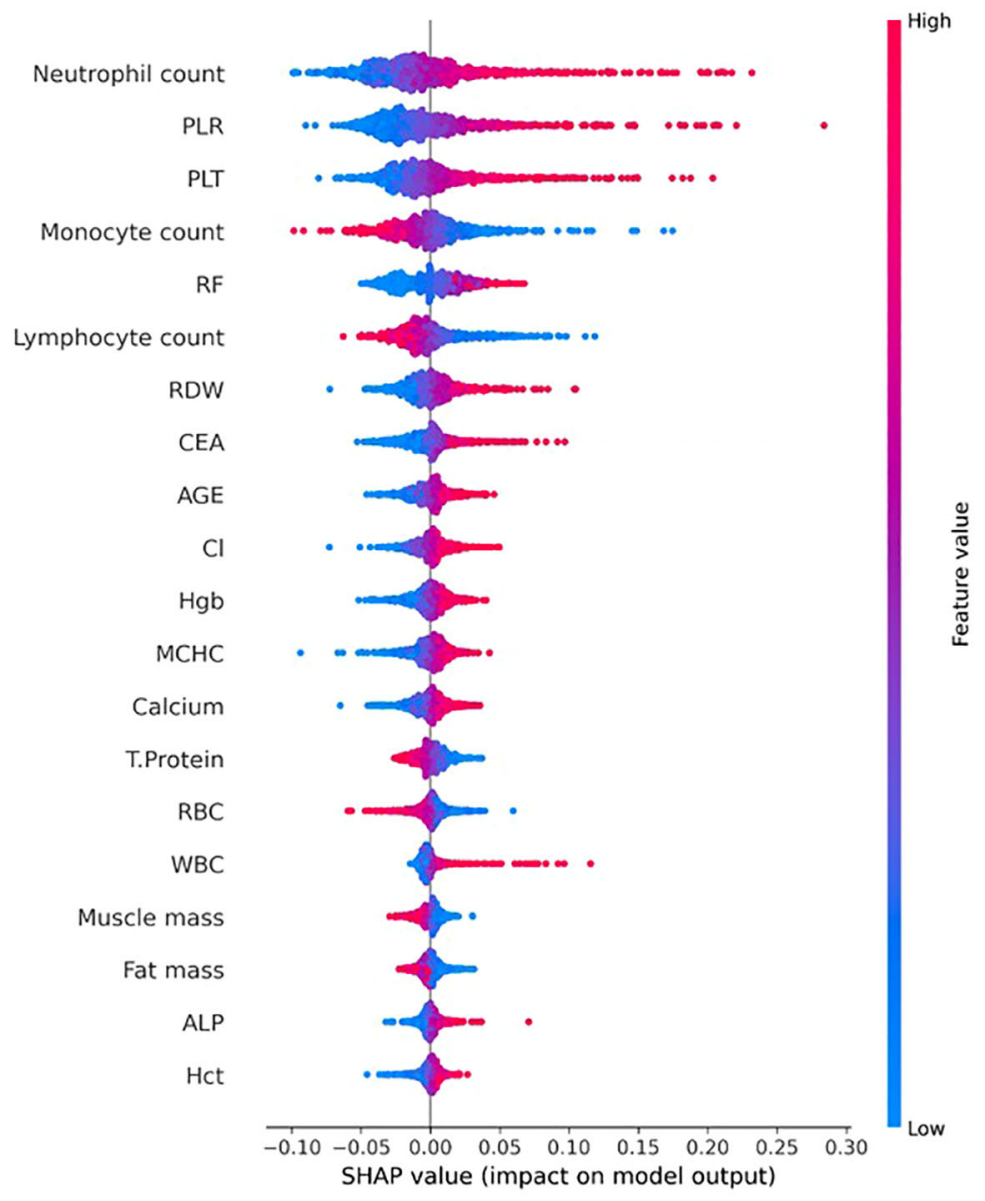
Top 20 most influential health-checkup variables identified from the TabPFN model with the best AUROC in our study.

**Figure 4** presents the top 20 health-checkup variables identified as the most influential in the SHAP analysis for the case of the best AUROC (i.e., the case of using TabPFN). All 20 items are consistent with our statistical analysis results, visualized in **Figures 1**, **2**. As shown in **Figure 4**, neutrophil count, PLR, and PLT were identified as the three most influential features. Higher values of neutrophil count, PLR, and PLT were associated with increased SHAP values toward the positive class (i.e., low immune status), indicating that elevated levels of these hematologic indicators contributed to predicting lower immune activity (Class 1).

## 4 Discussion

This study is the first to comprehensively analyze factors associated with NKA using AI and large-scale health checkup data from a single institution. By integrating clinical, biochemical, hematologic, and lifestyle information, this study provides an extensive overview of biological and behavioral factors influencing innate immune function in the general population. The AI-driven analysis identified multiple significant associations with NKA, encompassing hematologic inflammatory markers (NLR, PLR, platelet count, monocyte count), metabolic and biochemical indices (total protein, albumin, ALP, LDL, calcium, phosphorus), tumor markers (CEA), lifestyle factors (smoking, physical activity, sleep quality), and self-reported symptoms.

Individuals with lower NKA exhibited higher white blood cell (WBC), neutrophil, and platelet counts, as well as elevated NLR and PLR, whereas lymphocyte counts were lower. These findings reinforce the well-established link between systemic inflammation and immune suppression. Previous studies have shown that elevated NLR reflects chronic low-grade inflammation and impaired NK cytotoxicity (32–40). Similarly, platelets can suppress NK cell function via TGF-β1–dependent mechanisms, potentially facilitating tumor progression (34, 41). In addition, monocytes—another consistent correlating factor of NKA—exert both pro- and anti-inflammatory effects. Their cytokine signals can enhance NK activation under acute conditions but promote functional exhaustion during chronic inflammation, reflecting the bidirectional regulation between myeloid and innate immune pathways (42, 43).

The association between NKA and metabolic indices such as total protein, albumin, ALP, LDL, calcium, and phosphorus suggests a potential link between metabolic homeostasis and innate immunity. Low albumin and dyslipidemia are established markers of systemic inflammation and perturbed metabolic states (44, 45). NK cell effector function is tightly governed by cellular metabolism, and metabolic derangements particularly lipid overload and reduced metabolic flexibility impair NK cytotoxicity (46, 47). However, direct mechanistic evidence specifically connecting total protein, ALP, calcium, and phosphorus with NK cell activity is limited; these associations should therefore be interpreted cautiously and warrant targeted mechanistic study.

CEA, a well-known oncofetal glycoprotein expressed across various carcinomas, was another key predictor of reduced NKA. CEA can bind to inhibitory NK receptors and attenuate NK cell cytotoxicity independently of MHC class I recognition (48, 49). The observed association between elevated CEA levels and lower NKA suggests that diminished innate immune activity may facilitate tumor immune evasion and metastasis, particularly in cancers where NK cell–mediated cytotoxicity plays a central role, such as colorectal carcinoma (50).

Unlike previous NKA studies limited to laboratory data, our dataset incorporated extensive information on lifestyle and past medical history. Smoking was consistently associated with decreased NKA, whereas regular exercise and adequate sleep correlated with higher NKA. These findings align with prior evidence that nicotine exposure and oxidative stress suppress NK cell cytotoxicity and cytokine secretion (51) and that acute physical exercise robustly elevates NK-cell cytolytic activity (52). Sleep quality also emerged as a meaningful factor associated with NKA, consistent with accumulating evidence linking circadian rhythm and sleep disruption to NK cell trafficking and cytotoxic capacity (53, 54).

In addition, subjective symptoms—including fatigue, dizziness, nocturia, muscle pain, and heat intolerance—were inversely associated with NKA. These self-reported symptoms may reflect subclinical autonomic or neuroendocrine dysregulation, both of which are known to influence NK cell activity through stress-related signaling via the hypothalamic–pituitary–adrenal (HPA) axis and sympathetic pathways. Neuroendocrine mediators such as glucocorticoids and catecholamines have been shown to suppress NK cytotoxicity and cytokine production, highlighting the intimate link between neural and innate immune systems (42, 55). Collectively, these findings suggest that even mild physical complaints may serve as neuroimmune indicators of systemic immune vitality.

This study has several notable strengths. First, it is based on a large single-center cohort comprising over 11,000 participants. Second, it utilizes an integrated dataset that combines blood test results, anthropometric measurements, and lifestyle questionnaire data, allowing for a comprehensive analysis of immune activity. Third, the findings were consistent across multiple NKA thresholds, enhancing the robustness of the results. However, several limitations should be acknowledged. The study population consisted predominantly of Korean adults, which may limit the generalizability of the findings to other ethnic groups. In addition, the cross-sectional design precludes any causal inference between individual factors and immune activity. In addition, although AI-based NKA classification was feasible, discriminative performance was modest (best AUROC 0.716 ± 0.014), and the absence of external validation raises concerns about generalizability. Future work should incorporate multi-center, longitudinal validation and explore approaches that enhance representation and transferability—such as clinical large language models trained on electronic health records or biomedical texts to capture subtle semantic relationships among clinical variables (56, 57), and domain adaptation/generalization techniques to improve cross-dataset performance (e.g., differing patient populations and institutions) (58, 59).

## Data Availability

Data sharing is not applicable to this article, as the data cannot be shared publicly due to restrictions imposed by the ethics approval obtained from the institutional review board.
