# Supplementary material for: What affects natural killer cell activity: a cross-sectional study
Source: Front Immunol. 2026 Apr 29;17:1751240. doi: 10.3389/fimmu.2026.1751240 (PMC13167445; doi:10.3389/fimmu.2026.1751240)

# Supplementary Table 1. Clinical characteristics of the study population for total and three binary classification scenarios (unadjusted descriptive statistics)

| Variables | Total | Scenario 1 | | Scenario 2 | | Scenario 3 | |
| --- | --- | --- | --- | --- | --- | --- | --- |
|  |  | Low NKA Group (<100) | Non-low NKA Group ($\geq$100) | Low NKA Group (<250) | Non-low NKA Group ($\geq$250) | Low NKA Group (<500) | Non-low NKA Group ($\geq$500) |
| Participants, n | 11,007 | 1,109 | 9,898 | 2,452 | 8,555 | 4,142 | 6,865 |
| Age, years | 47.98 ±11.76 | 48.91 ±2.08 | 47.88 ±11.72 | 48.89 ±11.74 | 47.72 ±11.76 | 48.59 ±11.84 | 47.62 ±11.70 |
| Male sex, n (%) | 4,986 (45.3) | 428 (38.6) | 4,558 (46.0) | 1,051 (42.9) | 3,935 (46.0) | 1,869 (45.1) | 3,117 (45.4) |
| BMI, kg/m² | 23.33 ±3.76 | 22.95 ±3.81 | 23.37 ±3.75 | 23.25 ±3.98 | 23.35 ±3.70 | 23.34 ±3.94 | 23.33 ±3.65 |
| SBP, mmHg | 119.01 ±13.59 | 119.33 ±13.97 | 118.97 ±13.55 | 119.82 ±13.92 | 118.78 ±13.49 | 119.76 ±13.78 | 118.56 ±13.46 |
| DBP, mmHg | 77.30 ±10.96 | 77.63 ±10.93 | 77.26 ±10.96 | 77.95 ±11.13 | 77.11 ±10.91 | 77.89 ±11.09 | 76.94 ±10.87 |
| RHR, bpm | 74.39 ±11.57 | 78.02 ±12.73 | 73.99 ±11.36 | 76.57 ±12.32 | 73.77 ±11.26 | 75.90 ±12.16 | 73.49 ±11.10 |
| WBC, ×10³/µL | 5.49 ±1.59 | 6.41 ±2.23 | 5.38 ±1.47 | 6.05 ±1.97 | 5.32 ±1.42 | 5.88 ±1.82 | 5.25 ±1.38 |
| Neutrophil, ×10³/µL | 56.41 ±9.27 | 63.71 ±10.70 | 55.59 ±8.72 | 61.08 ±9.97 | 55.07 ±8.60 | 59.59 ±9.72 | 54.49 ±8.42 |
| Lymphocyte, ×10³/µL | 33.45 ±8.28 | 27.40 ±8.94 | 34.13 ±7.92 | 29.58 ±8.56 | 34.57 ±7.85 | 30.81 ±8.43 | 35.05 ±7.76 |
| Monocyte, ×10³/µL | 7.09 ±1.90 | 6.34 ±2.01 | 7.18 ±1.86 | 6.61 ±1.94 | 7.23 ±1.86 | 6.75 ±1.91 | 7.30 ±1.86 |
| Basophil, 10³/µL | 0.56 ±0.34 | 0.53 ±0.34 | 0.56 ±0.34 | 0.54 ±0.34 | 0.56 ±0.34 | 0.55 ±0.34 | 0.56 ±0.34 |
| Platelet, ×10³/µL | 253.44 ±57.41 | 277.44 ±69.28 | 250.75 ±55.28 | 269.98 ±64.39 | 248.70 ±54.33 | 265.22 ±62.12 | 246.33 ±53.12 |
| NLR | 1.91 ±1.07 | 2.88 ±2.02 | 1.80 ±0.84 | 2.45 ±1.59 | 1.75 ±0.80 | 2.26 ±1.39 | 1.70 ±0.75 |
| PLR | 8.23 ±3.67 | 11.69 ±6.45 | 7.84 ±2.97 | 10.25 ±5.24 | 7.65 ±2.83 | 9.53 ±4.60 | 7.45 ±2.69 |
| Total Cholesterol, mg/dL | 204.14 ±40.17 | 212.01 ±44.50 | 203.26 ±39.56 | 208.80 ±42.03 | 202.81 ±39.52 | 207.72 ±41.37 | 201.98 ±39.26 |
| ALP, U/L | 179.01 ±57.56 | 190.74 ±71.90 | 177.70 ±55.57 | 187.39 ±68.14 | 176.61 ±53.91 | 184.94 ±63.15 | 175.43 ±53.59 |
| Calcium, mg/dL | 9.19 ±0.40 | 9.24 ±0.43 | 9.18 ±0.40 | 9.23 ±0.42 | 9.18 ±0.40 | 9.22 ±0.41 | 9.17 ±0.40 |
| Phosphorus, mg/dL | 3.60 ±0.47 | 3.66 ±0.53 | 3.59 ±0.46 | 3.63 ±0.51 | 3.59 ±0.46 | 3.61 ±0.49 | 3.59 ±0.45 |
| CEA, ng/mL | 1.68 ±1.82 | 1.86 ±1.27 | 1.66 ±1.87 | 1.86 ±3.24 | 1.63 ±1.11 | 1.79 ±2.58 | 1.61 ±1.12 |
| Alcohol, n (%) | 7,584 (68.9) | 737 (66.5) | 6,847 (69.2) | 1,636 (66.7) | 5,948 (69.5) | 2,781 (67.1) | 4,803 (70.0) |
| Smoking, n (%) | 1,899 (17.3) | 222 (20.0) | 1,677 (16.9) | 457 (18.6) | 1,442 (16.9) | 770 (18.6) | 1,129 (16.4) |
| Regular exercise, n (%) | 7,201 (65.4) | 698 (62.9) | 6,503 (65.7) | 1,551 (63.3) | 5,650 (66.0) | 2,638 (63.7) | 4,563 (66.5) |
| Hypertension, n (%) | 1,583 (14.4) | 156 (14.1) | 1,427 (14.4) | 358 (14.6) | 1,225 (14.3) | 602 (14.5) | 981 (14.3) |
| Diabetes mellitus, n (%) | 599 (5.4) | 73 (6.6) | 526 (5.3) | 156 (6.4) | 443 (5.2) | 250 (6.0) | 349 (5.1) |
| Dyslipidemia, n (%) | 1,551 (14.1) | 161 (14.5) | 1,390 (14.0) | 367 (15.0) | 1,184 (13.8) | 588 (14.2) | 963 (14.0) |

Data are presented as mean ± SD or number (%). Group definitions: Group1 NKA <100 pg/mL; Group 2 NKA <250 pg/mL; Group3 NKA <500pg/mL.

Abbreviations: NKA, natural killer cell activity; BMI, body mass index; SBP, systolic blood pressure; DBP, diastolic blood pressure; RHR, Resting heart rate; WBC, white blood cell; NLR, neutrophil-to-lymphocyte ratio; PLR, platelet-to-lymphocyte ratio; ALP, alkaline phosphatase; CEA, carcinoembryonic antigen.

**Supplementary Table 2.** Univariable test results including raw p-values and false discovery rate (FDR)-adjusted q-values for binary NKA status using a threshold of 100 pg/mL. Variables and questionnaire items are arranged in ascending order of p-values within each category.

| **Category** | **Variable/Item** | **p-value** | **q-value** |
| --- | --- | --- | --- |
| Demographics | Sex | 0.000 | 0.000 |
|  | Age | 0.007 | 0.017 |
| Vital | RHR | 0.000 | 0.000 |
| Body composition | Height | 0.000 | 0.000 |
|  | Muscle mass | 0.000 | 0.000 |
|  | Muscle percentage | 0.000 | 0.000 |
|  | Fat percentage | 0.000 | 0.000 |
|  | Weight | 0.000 | 0.000 |
|  | BMI | 0.000 | 0.001 |
|  | Waist line | 0.008 | 0.019 |
| Hematologic indicators | WBC | 0.000 | 0.000 |
|  | RDW | 0.000 | 0.000 |
|  | PLT | 0.000 | 0.000 |
|  | Neutrophil count | 0.000 | 0.000 |
|  | Lymphocyte count | 0.000 | 0.000 |
|  | Monocyte count | 0.000 | 0.000 |
|  | Eosinophil count | 0.000 | 0.000 |
|  | NLR | 0.000 | 0.000 |
|  | PLR | 0.000 | 0.000 |
|  | MCHC | 0.001 | 0.002 |
|  | Basophil count | 0.001 | 0.003 |
| Metabolic & biochemical indicators | Total Protein | 0.000 | 0.000 |
|  | Albumin | 0.000 | 0.000 |
|  | Calcium | 0.000 | 0.000 |
|  | ALP | 0.000 | 0.000 |
|  | Total Cholesterol | 0.000 | 0.000 |
|  | LDL-C | 0.000 | 0.000 |
|  | Phosphorus | 0.000 | 0.000 |
|  | Creatinine | 0.000 | 0.000 |
|  | HDL-C | 0.000 | 0.001 |
|  | Estimated GFR | 0.006 | 0.015 |
|  | Cl | 0.018 | 0.039 |
|  | Uric acid | 0.021 | 0.044 |
| Immunological & inflammatory markers | CRP | 0.000 | 0.001 |
|  | ESR | 0.000 | 0.000 |
|  | RF | 0.036 | 0.067 |
| Tumor markers | CEA | 0.000 | 0.000 |
|  | CA 19-9 | 0.026 | 0.051 |
| Urine analysis | Urine protein | 0.000 | 0.000 |
|  | Urine pH | 0.000 | 0.000 |
| Medical history | Chronic pulmonary disease | 0.000 | 0.000 |
|  | Use of inhaled medications for asthma or chronic bronchitis | 0.002 | 0.004 |
|  | Depressive disorder or other psychiatric conditions | 0.020 | 0.043 |
|  | Family history of dementia | 0.025 | 0.050 |
|  | Family history of liver cancer | 0.035 | 0.067 |
|  | Family history of colorectal cancer | 0.045 | 0.080 |
| Lifestyle | Smoking | 0.009 | 0.023 |
|  | Daily activity intensity | 0.014 | 0.033 |
| Current symptom | Myalgia | 0.000 | 0.001 |
|  | Postnasal drip | 0.000 | 0.001 |
|  | Weight loss | 0.001 | 0.002 |
|  | Cold intolerance | 0.001 | 0.003 |
|  | Sputum production | 0.002 | 0.005 |
|  | Nocturia | 0.003 | 0.009 |
|  | Dizziness | 0.017 | 0.038 |
|  | Cutaneous allergic reactions or shortness of breath | 0.019 | 0.042 |
|  | Restricted joint range of motion | 0.020 | 0.043 |
|  | Paresthesia (tingling or abnormal sensations) | 0.024 | 0.049 |
|  | Joint swelling | 0.027 | 0.051 |
|  | Heat intolerance | 0.040 | 0.073 |
|  | Weight gain | 0.047 | 0.082 |

*p*-values were calculated using the t-test for continuous variables and the chi-square test for categorical variables. *q-*values were estimated using the Benjamini–Hochberg false discovery rate (FDR) procedure.

Abbreviations: FDR, false discovery rate; NKA, natural killer cell activity; RHR, Resting heart rate; BMI, body mass index; WBC, white blood cell; RDW, red cell distribution width; PLT, platelet count; NLR, neutrophil-to-lymphocyte ratio; PLR, platelet-to-lymphocyte ratio; MCHC, mean corpuscular hemoglobin concentration; ALP, alkaline phosphatase; LDL-C, low-density lipoprotein cholesterol; HDL-C, high-density lipoprotein cholesterol; GFR, glomerular filtration rate; Cl, chloride; CRP, C-reactive protein; ESR, erythrocyte sedimentation rate; RF, rheumatoid factor; CEA, carcinoembryonic antigen; CA 19-9, carbohydrate antigen 19-9.

**Supplementary Table 3.** Univariable test results including raw p-values and false discovery rate (FDR)-adjusted q-values for binary NKA status using a threshold of 250 pg/mL. Variables and questionnaire items are arranged in ascending order of p-values within each category.

| **Category** | **Variable/Item** | **p-value** | **q-value** |
| --- | --- | --- | --- |
| Demographics | Age | 0.000 | 0.000 |
|  | Sex | 0.006 | 0.017 |
| Vital | RHR | 0.000 | 0.000 |
|  | SBP | 0.001 | 0.003 |
|  | DBP | 0.001 | 0.003 |
| Body composition | Height | 0.000 | 0.000 |
|  | Muscle mass | 0.000 | 0.000 |
|  | Muscle percentage | 0.000 | 0.000 |
|  | Fat percentage | 0.000 | 0.000 |
|  | Weight | 0.000 | 0.000 |
|  | WHR | 0.014 | 0.036 |
|  | Fat mass | 0.035 | 0.067 |
| Hematologic indicators | WBC | 0.000 | 0.000 |
|  | RDW | 0.000 | 0.000 |
|  | PLT | 0.000 | 0.000 |
|  | Neutrophil count | 0.000 | 0.000 |
|  | Lymphocyte count | 0.000 | 0.000 |
|  | Monocyte count | 0.000 | 0.000 |
|  | Eosinophil count | 0.000 | 0.000 |
|  | NLR | 0.000 | 0.000 |
|  | PLR | 0.000 | 0.000 |
|  | MCHC | 0.001 | 0.003 |
|  | Hct | 0.001 | 0.003 |
|  | RBC | 0.004 | 0.012 |
|  | Basophil count | 0.018 | 0.041 |
|  | Hgb | 0.027 | 0.053 |
| Metabolic & biochemical indicators | Total Protein | 0.000 | 0.000 |
|  | Albumin | 0.000 | 0.000 |
|  | Calcium | 0.000 | 0.000 |
|  | ALP | 0.000 | 0.000 |
|  | Total Cholesterol | 0.000 | 0.000 |
|  | LDL-C | 0.000 | 0.000 |
|  | Cl | 0.000 | 0.000 |
|  | Phosphorus | 0.001 | 0.002 |
|  | Triglyceride | 0.007 | 0.019 |
|  | Creatinine | 0.015 | 0.036 |
| Immunological & inflammatory markers | CRP | 0.000 | 0.000 |
|  | ESR | 0.000 | 0.000 |
|  | RF | 0.017 | 0.040 |
|  | VDRL | 0.022 | 0.048 |
| Tumor markers | CEA | 0.001 | 0.002 |
|  | CA 19-9 | 0.014 | 0.036 |
| Urine analysis | Urine protein | 0.000 | 0.000 |
|  | Urine pH | 0.000 | 0.000 |
|  | Urine specific gravity | 0.047 | 0.085 |
| Medication | Antidiabetic medication | 0.027 | 0.053 |
| Medical history | Family history of colorectal cancer | 0.022 | 0.048 |
|  | Use of inhaled medications for asthma or chronic bronchitis | 0.024 | 0.050 |
|  | Benign prostatic hyperplasia | 0.024 | 0.050 |
|  | Asthma | 0.027 | 0.053 |
| Lifestyle | Alcohol consumption | 0.008 | 0.022 |
|  | Exercise | 0.015 | 0.036 |
|  | Smoking | 0.037 | 0.069 |
| Current symptom | Weight loss | 0.000 | 0.000 |
|  | Myalgia | 0.001 | 0.003 |
|  | Heat intolerance | 0.003 | 0.008 |
|  | Nocturia | 0.004 | 0.013 |
|  | Postnasal drip | 0.017 | 0.040 |
|  | Facial flushing | 0.037 | 0.069 |
|  | Dizziness | 0.045 | 0.083 |

*p*-values were calculated using the t-test for continuous variables and the chi-square test for categorical variables. *q-*values were estimated using the Benjamini–Hochberg false discovery rate (FDR) procedure.

Abbreviation: FDR, false discovery rate; NKA, natural killer cell activity; RHR, Resting heart rate; SBP, systolic blood pressure; DBP, diastolic blood pressure; WHR, waist-to-height ratio; WBC, white blood cell; RDW, red cell distribution width; PLT, platelet count; NLR, neutrophil-to-lymphocyte ratio; PLR, platelet-to-lymphocyte ratio; MCHC, mean corpuscular hemoglobin concentration; Hct, hematocrit; RBC, red blood cell count; Hgb, hemoglobin; ALP, alkaline phosphatase; LDL-C, low-density lipoprotein cholesterol; Cl, chloride; CRP, C-reactive protein; ESR, erythrocyte sedimentation rate; RF, rheumatoid factor; VDRL, Venereal Disease Research Laboratory test; CEA, carcinoembryonic antigen; CA 19-9, carbohydrate antigen 19-9.

**Supplementary Table 4.** Univariable test results including raw p-values and false discovery rate (FDR)-adjusted q-values for binary NKA status using a threshold of 500 pg/mL. Variables and questionnaire items are arranged in ascending order of p-values within each category.

| **Category** | **Variable/Item** | **p-value** | **q-value** |
| --- | --- | --- | --- |
| Demographics | Age | 0.000 | 0.000 |
| Vital | RHR | 0.000 | 0.000 |
|  | SBP | 0.000 | 0.000 |
|  | DBP | 0.000 | 0.000 |
| Body composition | Height | 0.000 | 0.000 |
|  | Muscle mass | 0.000 | 0.000 |
|  | Muscle percentage | 0.000 | 0.000 |
|  | Fat percentage | 0.000 | 0.000 |
|  | Weight | 0.035 | 0.066 |
|  | WHR | 0.000 | 0.001 |
|  | Fat mass | 0.004 | 0.010 |
| Hematologic indicators | WBC | 0.000 | 0.000 |
|  | RDW | 0.000 | 0.000 |
|  | PLT | 0.000 | 0.000 |
|  | Neutrophil count | 0.000 | 0.000 |
|  | Lymphocyte count | 0.000 | 0.000 |
|  | Monocyte count | 0.000 | 0.000 |
|  | Eosinophil count | 0.000 | 0.000 |
|  | NLR | 0.000 | 0.000 |
|  | PLR | 0.000 | 0.000 |
|  | MCHC | 0.008 | 0.019 |
|  | Basophil count | 0.027 | 0.053 |
|  | RBC | 0.000 | 0.000 |
|  | Hct | 0.000 | 0.000 |
|  | Hgb | 0.000 | 0.000 |
| Metabolic & biochemical indicators | Total Protein | 0.000 | 0.000 |
|  | Albumin | 0.000 | 0.000 |
|  | Calcium | 0.000 | 0.000 |
|  | ALP | 0.000 | 0.000 |
|  | Total Cholesterol | 0.000 | 0.000 |
|  | LDL-C | 0.000 | 0.000 |
|  | Phosphorus | 0.010 | 0.023 |
|  | Cl | 0.000 | 0.000 |
|  | Triglyceride | 0.000 | 0.000 |
|  | Estimated GFR | 0.049 | 0.084 |
|  | Total Bilirubin | 0.011 | 0.025 |
|  | GGT | 0.019 | 0.039 |
| Immunological & inflammatory markers | CRP | 0.000 | 0.000 |
|  | ESR | 0.000 | 0.000 |
|  | RF | 0.000 | 0.001 |
| Tumor markers | CEA | 0.000 | 0.000 |
|  | AFP | 0.045 | 0.081 |
| Urine analysis | Urine protein | 0.000 | 0.000 |
|  | Urine pH | 0.000 | 0.000 |
|  | Urine specific gravity | 0.006 | 0.016 |
| Medication | Antidiabetic medication | 0.036 | 0.068 |
| Medical history | Use of inhaled medications for asthma or chronic bronchitis | 0.026 | 0.052 |
|  | Arrhythmia | 0.007 | 0.016 |
|  | History of lumbar disc surgery | 0.023 | 0.048 |
|  | Family history of colorectal cancer | 0.040 | 0.074 |
| Lifestyle | Smoking | 0.014 | 0.030 |
|  | Alcohol consumption | 0.002 | 0.005 |
|  | Daily activity intensity | 0.008 | 0.019 |
|  | Exercise | 0.005 | 0.013 |
| Current symptom | Weight loss | 0.000 | 0.000 |
|  | Myalgia | 0.001 | 0.002 |
|  | Nocturia | 0.006 | 0.015 |
|  | Dizziness | 0.002 | 0.005 |
|  | Heat intolerance | 0.046 | 0.081 |
|  | Oral vesicles or ulcers (lips, gums, tongue) | 0.007 | 0.016 |
|  | Diplopia (double vision) | 0.048 | 0.083 |
|  | Dyspepsia | 0.022 | 0.046 |
|  | Alopecia | 0.025 | 0.049 |

*p*-values were calculated using the t-test for continuous variables and the chi-square test for categorical variables. *q-*values were estimated using the Benjamini–Hochberg false discovery rate (FDR) procedure.

Abbreviation: FDR, false discovery rate; NKA, natural killer cell activity; RHR, Resting heart rate; SBP, systolic blood pressure; DBP, diastolic blood pressure; WBC, white blood cell; RDW, red cell distribution width; PLT, platelet count; NLR, neutrophil-to-lymphocyte ratio; PLR, platelet-to-lymphocyte ratio; MCHC, mean corpuscular hemoglobin concentration; RBC, red blood cell count; Hct, hematocrit; Hgb, hemoglobin; ALP, alkaline phosphatase; LDL-C, low-density lipoprotein cholesterol; Cl, chloride; GFR, glomerular filtration rate; GGT, gamma-glutamyl transferase; CRP, C-reactive protein; ESR, erythrocyte sedimentation rate; RF, rheumatoid factor; CEA, carcinoembryonic antigen; AFP, alpha-fetoprotein.

**Supplementary Table 5.** Multivariate logistic regression results for binary NKA status using a threshold of 100 pg/mL. Variables and questionnaire items are arranged in ascending order of p-values within each category.

| **Category** | **Variable/Item** | **p-value** | **OR** | **CI low** | **CI high** |  |
| --- | --- | --- | --- | --- | --- | --- |
| Vital | RHR | 0.000 | 1.029 | 1.023 | 1.034 |  |
| Body composition | Muscle mass | 0.000 | 0.852 | 0.798 | 0.909 |  |
|  | Waist line | 0.011 | 1.022 | 1.005 | 1.040 |  |
|  | Muscle percentage | 0.013 | 1.128 | 1.026 | 1.240 |  |
|  | Weight | 0.027 | 1.085 | 1.009 | 1.166 |  |
|  | Fat percentage | 0.090 | 1.074 | 0.989 | 1.167 |  |
|  | Height | 0.760 | 0.993 | 0.946 | 1.041 |  |
| Hematologic indicators | PLT | 0.000 | 1.009 | 1.006 | 1.012 |  |
|  | NLR | 0.001 | 1.431 | 1.160 | 1.765 |  |
|  | MCHC | 0.001 | 1.149 | 1.057 | 1.250 |  |
|  | WBC | 0.002 | 1.080 | 1.029 | 1.133 |  |
|  | RDW | 0.033 | 1.084 | 1.007 | 1.167 |  |
|  | Monocyte count | 0.042 | 0.815 | 0.669 | 0.992 |  |
|  | PLR | 0.102 | 0.943 | 0.879 | 1.012 |  |
|  | Lymphocyte count | 0.132 | 0.862 | 0.711 | 1.046 |  |
|  | Basophil count | 0.161 | 1.206 | 0.928 | 1.566 |  |
|  | Eosinophil count | 0.243 | 0.889 | 0.730 | 1.083 |  |
|  | Neutrophil count | 0.370 | 0.916 | 0.755 | 1.110 |  |
| Metabolic  & biochemical indicators | ALP | 0.000 | 1.002 | 1.001 | 1.004 |  |
|  | Total Protein | 0.001 | 1.429 | 1.163 | 1.756 |  |
|  | Estimated GFR | 0.007 | 1.011 | 1.003 | 1.020 |  |
|  | Cl | 0.068 | 1.023 | 0.998 | 1.048 |  |
|  | Total Cholesterol | 0.090 | 1.004 | 0.999 | 1.008 |  |
|  | Creatinine | 0.308 | 1.639 | 0.634 | 4.238 |  |
|  | Phosphorus | 0.639 | 0.965 | 0.831 | 1.121 |  |
|  | Uric acid | 0.673 | 0.987 | 0.927 | 1.050 |  |
|  | HDL-C | 0.738 | 1.001 | 0.995 | 1.007 |  |
|  | Albumin | 0.803 | 1.044 | 0.744 | 1.466 |  |
|  | Calcium | 0.818 | 1.024 | 0.836 | 1.255 |  |
|  | LDL-C | 0.937 | 1.000 | 0.995 | 1.004 |  |
| Immunological & inflammatory markers | ESR | 0.000 | 1.026 | 1.019 | 1.032 |  |
|  | CRP | 0.034 | 1.160 | 1.011 | 1.330 |  |
|  | RF | 0.328 | 1.002 | 0.998 | 1.007 |  |
| Tumor markers | CA 19-9 | 0.010 | 1.008 | 1.002 | 1.014 |  |
|  | CEA | 0.108 | 1.030 | 0.994 | 1.068 |  |
| Urine analysis | Urine protein | 0.000 | 1.578 | 1.300 | 1.915 |  |
|  | Urine pH | 0.000 | 0.829 | 0.767 | 0.896 |  |
| Medical history | Family history of dementia | 0.006 | 0.745 | 0.604 | 0.920 |  |
|  | Chronic pulmonary disease | 0.007 | 2.909 | 1.342 | 6.307 |  |
|  | Use of inhaled medications for asthma or chronic bronchitis | 0.009 | 2.110 | 1.206 | 3.692 |  |
|  | Family history of liver cancer | 0.033 | 0.723 | 0.538 | 0.973 |  |
|  | Depressive disorder or other psychiatric conditions | 0.051 | 1.342 | 0.999 | 1.804 |  |
|  | Family history of colorectal cancer | 0.060 | 1.250 | 0.990 | 1.578 |  |
| Lifestyle | Daily activity intensity (moderate; 4 out of 5 levels) | 0.004 | 0.538 | 0.353 | 0.820 |  |
|  | Daily activity intensity (sedentary; 2 out of 5 levels) | 0.018 | 0.635 | 0.436 | 0.924 |  |
|  | Daily activity intensity (light; 3 out of 5 levels) | 0.044 | 0.669 | 0.453 | 0.989 |  |
|  | Daily activity intensity (vigorous; 5 out of 5 levels) | 0.406 | 0.740 | 0.365 | 1.503 |  |
| Current symptom | Postnasal drip | 0.010 | 1.387 | 1.080 | 1.782 |  |
|  | Myalgia | 0.017 | 1.245 | 1.039 | 1.491 |  |
|  | Weight gain | 0.032 | 0.822 | 0.687 | 0.983 |  |
|  | Sputum production | 0.047 | 1.237 | 1.003 | 1.526 |  |
|  | Weight loss | 0.058 | 1.349 | 0.990 | 1.837 |  |
|  | Nocturia | 0.181 | 1.148 | 0.938 | 1.406 |  |
|  | Restricted joint range of motion | 0.183 | 1.244 | 0.902 | 1.716 |  |
|  | Cold intolerance | 0.271 | 1.107 | 0.924 | 1.327 |  |
|  | Cutaneous allergic reactions or shortness of breath | 0.275 | 1.130 | 0.907 | 1.408 |  |
|  | Joint swelling | 0.418 | 1.187 | 0.784 | 1.796 |  |
|  | Heat intolerance | 0.669 | 1.049 | 0.842 | 1.307 |  |
|  | Dizziness | 0.805 | 1.025 | 0.843 | 1.246 |  |
|  | Paresthesia (tingling or abnormal sensations) | 0.954 | 1.007 | 0.799 | 1.268 |  |

*p*-value, OR, CI low, and CI high were calculated using the multivariate logistic regression where covariates were age, sex, BMI, drinking status, smoking status, and self-reported use of antihypertensive, antidiabetic, and lipid-lowering medications.

Abbreviations: NKA, natural killer cell activity; OR, odds ratio; CI, confidence interval; RHR, Resting heart rate; PLT, platelet count; NLR, neutrophil-to-lymphocyte ratio; MCHC, mean corpuscular hemoglobin concentration; WBC, white blood cell; RDW, red cell distribution width; PLR, platelet-to-lymphocyte ratio; ALP, alkaline phosphatase; GFR, glomerular filtration rate; Cl, chloride; HDL-C, high-density lipoprotein cholesterol; LDL-C, low-density lipoprotein cholesterol; ESR, erythrocyte sedimentation rate; CRP, C-reactive protein; RF, rheumatoid factor; CA 19-9, carbohydrate antigen 19-9; CEA, carcinoembryonic antigen; BMI, body mass index.

**Supplementary Table 6.** Multivariate logistic regression results for binary NKA status using a threshold of 500 pg/mL. Variables and questionnaire items are arranged in ascending order of p-values within each category.

| **Category** | **Variable/Item** | **p-value** | **OR** | **CI low** | **CI high** |
| --- | --- | --- | --- | --- | --- |
| Vital | RHR | 0.000 | 1.018 | 1.015 | 1.022 |
|  | SBP | 0.011 | 1.006 | 1.001 | 1.011 |
|  | DBP | 0.664 | 0.999 | 0.993 | 1.005 |
| Body composition | WHR | 0.001 | 19.668 | 3.314 | 116.715 |
|  | Fat mass | 0.170 | 1.999 | 0.743 | 5.381 |
|  | Fat percentage | 0.213 | 0.688 | 0.381 | 1.240 |
|  | Weight | 0.225 | 0.544 | 0.203 | 1.455 |
|  | Muscle mass | 0.270 | 1.799 | 0.634 | 5.111 |
|  | Muscle percentage | 0.292 | 0.715 | 0.384 | 1.333 |
|  | Height | 0.793 | 1.004 | 0.974 | 1.034 |
| Hematologic indicators | RDW | 0.000 | 1.212 | 1.144 | 1.283 |
|  | PLT | 0.000 | 1.007 | 1.005 | 1.010 |
|  | RBC | 0.000 | 0.589 | 0.473 | 0.735 |
|  | NLR | 0.001 | 1.443 | 1.159 | 1.796 |
|  | Basophil count | 0.007 | 1.231 | 1.057 | 1.434 |
|  | WBC | 0.016 | 1.043 | 1.008 | 1.080 |
|  | PLR | 0.094 | 0.942 | 0.877 | 1.010 |
|  | Monocyte count | 0.109 | 0.918 | 0.827 | 1.019 |
|  | Hgb | 0.324 | 1.482 | 0.678 | 3.240 |
|  | Neutrophil count | 0.622 | 1.026 | 0.927 | 1.135 |
|  | Hct | 0.720 | 0.954 | 0.736 | 1.235 |
|  | Lymphocyte count | 0.802 | 0.987 | 0.891 | 1.093 |
|  | MCHC | 0.858 | 0.971 | 0.703 | 1.341 |
|  | Eosinophil count | 0.945 | 1.004 | 0.904 | 1.114 |
| Metabolic  & biochemical indicators | ALP | 0.000 | 1.002 | 1.001 | 1.003 |
|  | Cl | 0.000 | 1.048 | 1.032 | 1.065 |
|  | Triglyceride | 0.000 | 1.001 | 1.001 | 1.002 |
|  | Estimated GFR | 0.000 | 1.008 | 1.005 | 1.010 |
|  | Calcium | 0.001 | 1.247 | 1.096 | 1.417 |
|  | Total Bilirubin | 0.002 | 1.200 | 1.067 | 1.349 |
|  | Total Protein | 0.079 | 1.124 | 0.986 | 1.282 |
|  | LDL-C | 0.169 | 1.002 | 0.999 | 1.005 |
|  | Phosphorus | 0.324 | 0.954 | 0.868 | 1.048 |
|  | GGT | 0.427 | 1.000 | 0.999 | 1.001 |
|  | Albumin | 0.502 | 1.076 | 0.869 | 1.333 |
|  | Total Cholesterol | 0.847 | 1.000 | 0.998 | 1.003 |
| Immunological  & inflammatory markers | ESR | 0.000 | 1.020 | 1.015 | 1.025 |
|  | RF | 0.004 | 1.005 | 1.001 | 1.008 |
|  | CRP | 0.015 | 1.189 | 1.035 | 1.366 |
| Tumor markers | CEA | 0.000 | 1.098 | 1.058 | 1.140 |
|  | AFP | 0.011 | 1.021 | 1.005 | 1.037 |
| Urine analysis | Urine protein | 0.000 | 1.640 | 1.429 | 1.883 |
|  | Urine pH | 0.000 | 0.870 | 0.830 | 0.912 |
|  | Urine specific gravity | 0.563 | 5.266 | 0.019 | 1461.036 |
| Medical history | Arrhythmia | 0.002 | 0.667 | 0.516 | 0.864 |
|  | History of lumbar disc surgery | 0.008 | 0.693 | 0.530 | 0.908 |
|  | Family history of colorectal cancer | 0.027 | 1.189 | 1.020 | 1.387 |
|  | Use of inhaled medications for asthma or chronic bronchitis | 0.030 | 1.620 | 1.049 | 2.502 |
| Lifestyle | Exercise (4+/week) | 0.002 | 0.782 | 0.669 | 0.914 |
|  | Daily activity intensity (light; 3 out of 5 levels) | 0.003 | 0.647 | 0.487 | 0.860 |
|  | Daily activity intensity (sedentary; 2 out of 5 levels) | 0.006 | 0.680 | 0.517 | 0.896 |
|  | Daily activity intensity (moderate; 4 out of 5 levels) | 0.012 | 0.680 | 0.503 | 0.918 |
|  | Exercise (2~4/week) | 0.030 | 0.893 | 0.806 | 0.989 |
|  | Daily activity intensity (vigorous; 5 out of 5 levels) | 0.247 | 0.754 | 0.467 | 1.216 |
|  | Exercise (2~4/month) | 0.311 | 0.939 | 0.833 | 1.060 |
| Current symptom | Weight loss | 0.001 | 1.438 | 1.153 | 1.793 |
|  | Myalgia | 0.001 | 1.216 | 1.078 | 1.371 |
|  | Oral vesicles or ulcers (lips, gums, tongue) | 0.002 | 0.759 | 0.640 | 0.901 |
|  | Dyspepsia | 0.002 | 0.846 | 0.763 | 0.938 |
|  | Dizziness | 0.006 | 1.196 | 1.054 | 1.358 |
|  | Diplopia (double vision) | 0.006 | 0.685 | 0.523 | 0.897 |
|  | Alopecia | 0.089 | 1.100 | 0.985 | 1.229 |
|  | Heat intolerance | 0.121 | 1.120 | 0.970 | 1.294 |
|  | Nocturia | 0.159 | 1.101 | 0.963 | 1.258 |

*p*-value, OR, CI low, and CI high were calculated using the multivariate logistic regression where covariates were age, sex, BMI, drinking status, smoking status, and self-reported use of antihypertensive, antidiabetic, and lipid-lowering medications.

Abbreviations: NKA, natural killer cell activity; OR, odds ratio; CI, confidence interval; RHR, Resting heart rate; SBP, systolic blood pressure; DBP, diastolic blood pressure; WHR, waist-to-height ratio; RDW, red cell distribution width; PLT, platelet count; RBC, red blood cell count; NLR, neutrophil-to-lymphocyte ratio; WBC, white blood cell; PLR, platelet-to-lymphocyte ratio; Hgb, hemoglobin; Hct, hematocrit; MCHC, mean corpuscular hemoglobin concentration; ALP, alkaline phosphatase; Cl, chloride; GFR, glomerular filtration rate; LDL-C, low-density lipoprotein cholesterol; GGT, gamma-glutamyl transferase; ESR, erythrocyte sedimentation rate; RF, rheumatoid factor; CRP, C-reactive protein; CEA, carcinoembryonic antigen; AFP, alpha-fetoprotein; BMI, body mass index.**Supplementary Table 7.** Age and sex distribution across the data of 5 folds used in our AI-based NKA classification experiments

| **Category** | | **Fold 1** | **Fold 2** | **Fold 3** | **Fold 4** | **Fold 5** |
| --- | --- | --- | --- | --- | --- | --- |
| **Age** |  |  | | | | |
|  | <20, n | 9 | 9 | 8 | 9 | 9 |
|  | 20-29, n | 116 | 118 | 117 | 116 | 116 |
|  | 30-39, n | 410 | 410 | 409 | 409 | 410 |
|  | 40-49, n | 694 | 693 | 693 | 693 | 692 |
|  | 50-59, n | 622 | 621 | 621 | 621 | 622 |
|  | 60-69, n | 254 | 254 | 254 | 255 | 254 |
|  | 70-79, n | 87 | 88 | 89 | 88 | 88 |
|  | 80$\geq$, n | 10 | 9 | 10 | 10 | 10 |
| **Sex** |  |  | | | | |
|  | Female, n | 1,206 | 1,205 | 1,204 | 1,202 | 1,204 |
|  | Male, n | 996 | 997 | 997 | 999 | 997 |

**Supplementary Table 8.** NKA binary classification results for nine AI models using alternative thresholds of 100 pg/mL and 500 pg/mL

| **Scenario** | **AI model** | **AUROC** | **PRAUC** | **Accuracy** | **Precision** | **Recall** | **Specificity** | **F1 score** |
| --- | --- | --- | --- | --- | --- | --- | --- | --- |
| “NKA<100” Group  vs.  “NKA≥250” Group | Linear Regression | 0.758 (±0.014) | 0.335 | 0.747 | 0.241 | 0.625 | 0.762 | 0.338 |
|  | Random Forest | 0.747 (±0.016) | 0.335 | 0.768 | 0.243 | 0.585 | 0.788 | 0.339 |
|  | XGBoost | 0.745 (±0.012) | 0.324 | 0.712 | 0.208 | 0.658 | 0.718 | 0.315 |
|  | CatBoost | 0.757 (±0.014) | 0.342 | 0.752 | 0.231 | 0.630 | 0.766 | 0.338 |
|  | FCNN | **0.762 (±0.011)** | 0.333 | 0.716 | 0.214 | 0.679 | 0.720 | 0.325 |
|  | TabNet | 0.755 (±0.017) | 0.327 | 0.720 | 0.222 | 0.664 | 0.726 | 0.326 |
|  | FT‑Transformer | 0.754 (±0.013) | 0.330 | 0.770 | 0.246 | 0.594 | 0.790 | 0.344 |
|  | TabPFN | 0.756 (±0.017) | 0.347 | 0.781 | 0.252 | 0.584 | 0.803 | 0.349 |
|  | ExcelFormer | 0.754 (±0.016) | 0.332 | 0.728 | 0.218 | 0.647 | 0.737 | 0.325 |
|  | TabM | 0.761 (±0.014) | 0.338 | 0.733 | 0.231 | 0.655 | 0.743 | 0.335 |
| “NKA<500” Group  vs.  “NKA≥500” Group | Linear Regression | 0.700 (±0.011) | 0.602 | 0.666 | 0.552 | 0.593 | 0.708 | 0.571 |
|  | Random Forest | 0.688 (±0.009) | 0.585 | 0.649 | 0.534 | 0.600 | 0.676 | 0.561 |
|  | XGBoost | 0.689 (±0.008) | 0.587 | 0.649 | 0.531 | 0.609 | 0.674 | 0.566 |
|  | CatBoost | 0.698 (±0.010) | 0.600 | 0.661 | 0.551 | 0.586 | 0.708 | 0.565 |
|  | FCNN | 0.699 (±0.010) | 0.600 | 0.653 | 0.535 | 0.614 | 0.677 | 0.571 |
|  | TabNet | 0.694 (±0.009) | 0.593 | 0.654 | 0.536 | 0.612 | 0.679 | 0.57 |
|  | FT‑Transformer | 0.697 (±0.008) | 0.595 | 0.645 | 0.523 | 0.657 | 0.636 | 0.582 |
|  | TabPFN | **0.703 (±0.011)** | 0.605 | 0.666 | 0.553 | 0.594 | 0.710 | 0.571 |
|  | ExcelFormer | 0.699 (±0.011) | 0.596 | 0.656 | 0.540 | 0.614 | 0.680 | 0.573 |
|  | TabM | 0.699 (±0.011) | 0.600 | 0.660 | 0.543 | 0.601 | 0.695 | 0.570 |

**Supplementary Figure 1.** Heatmap illustrating the statistical significance of 54 health examination variables for NKA binary classification (thresholds: 100 and 500, respectively). The cells are colored white (non-significant) or in three varying intensities of red based on their respective p-values.

**
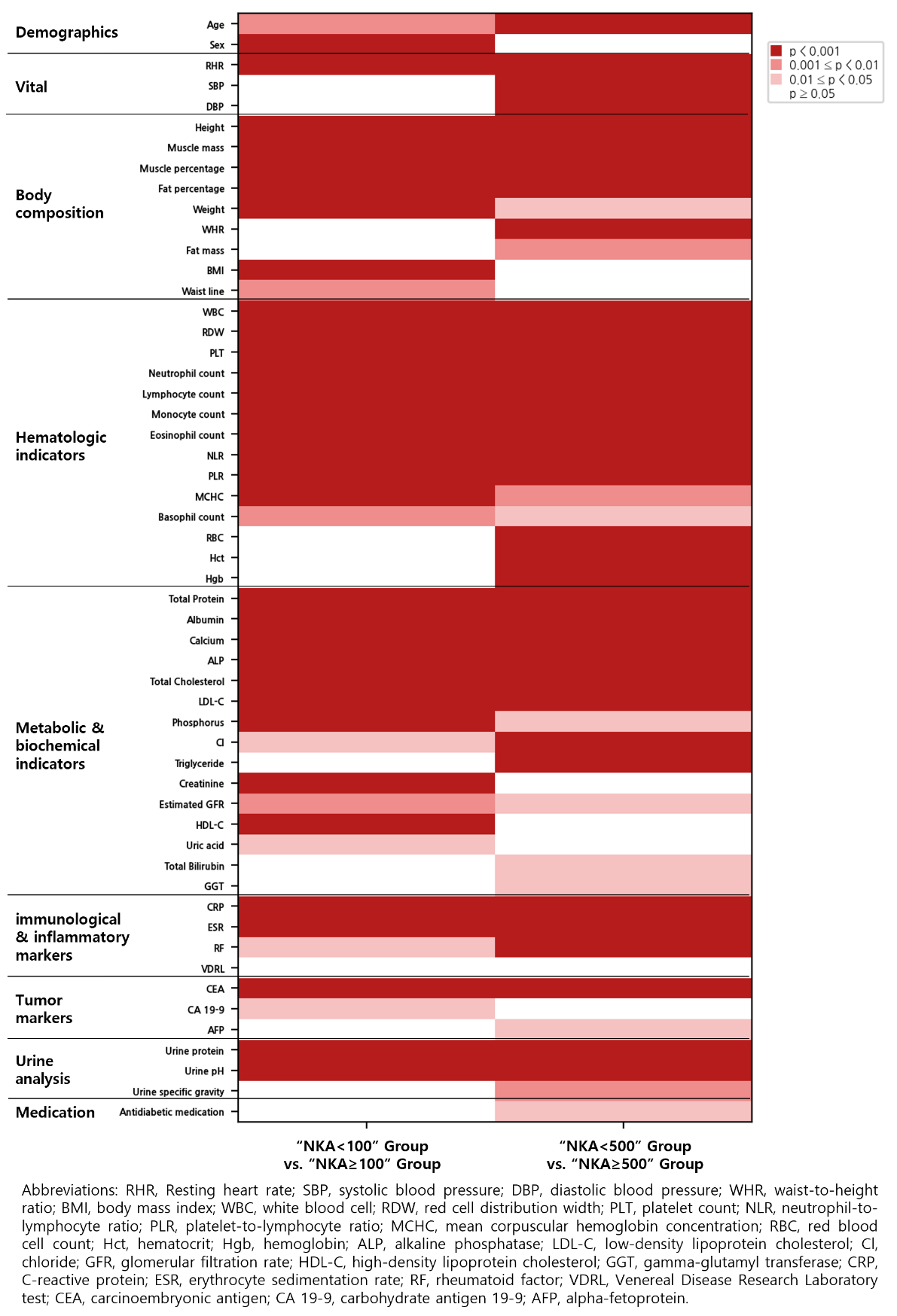
**

**Supplementary Figure 2.** Heatmap illustrating the statistical significance of 32 questionnaire items for NKA binary classification (thresholds: 100 and 500, respectively). The cells are colored white (non-significant) or in three varying intensities of red based on their respective p-values.


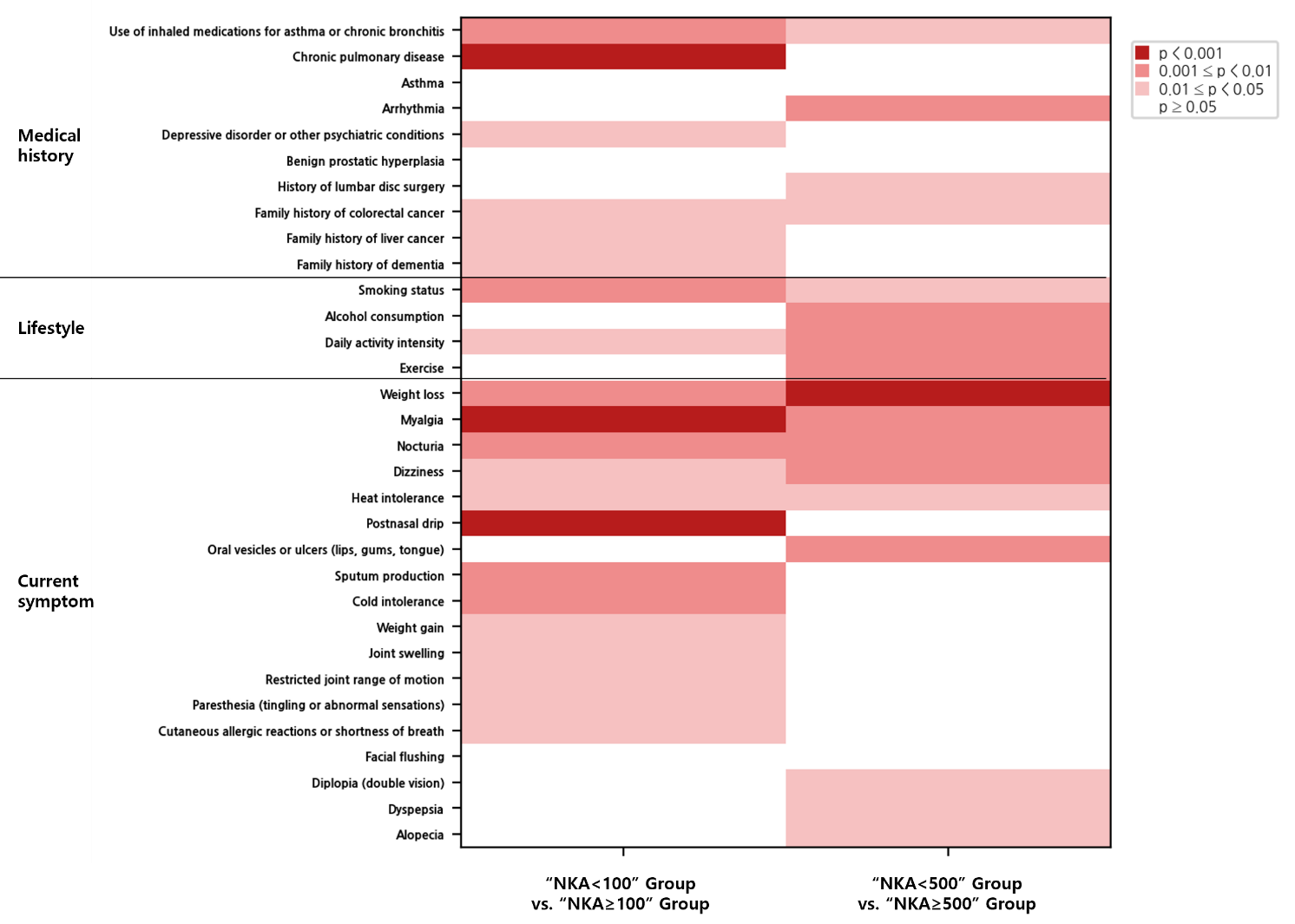


**Supplementary Figure 3.** IQR plots of the statistically significant numerical variables according to binary NKA status (threshold: 100). Variables were grouped into eight categories which are demographics, vital, body composition, hematologic indicators, metabolic & biochemical indicators, immunological & inflammatory markers, tumor markers, and urine analysis. X-axis represents z-score-normalized values. The median value of each variable for each group was annotated as a diamond-shaped figure on the bar.


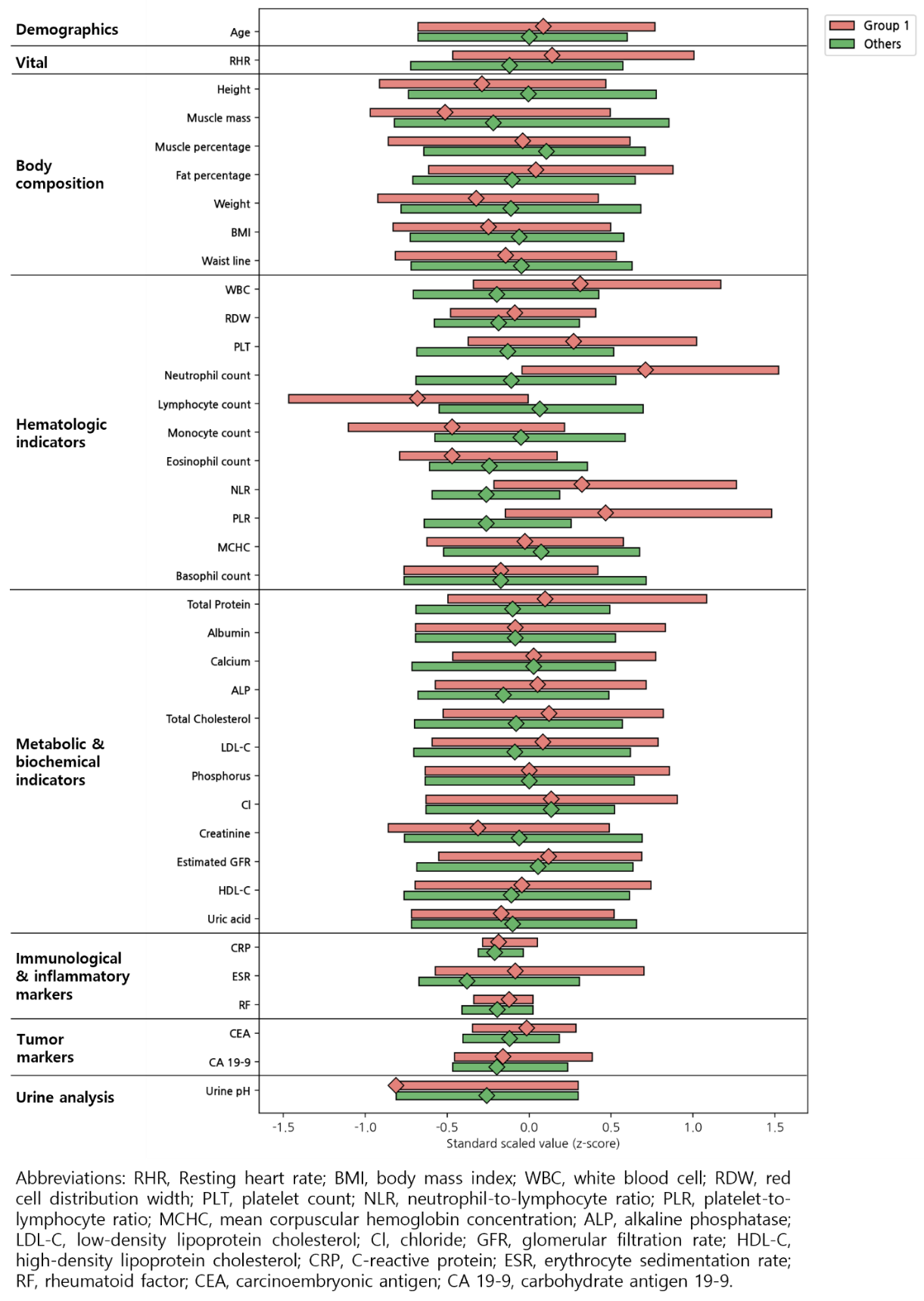


**Supplementary Figure 4.** IQR plots of the statistically significant numerical variables according to binary NKA status (threshold: 250). Variables were grouped into eight categories which are demographics, vital, body composition, hematologic indicators, metabolic & biochemical indicators, immunological & inflammatory markers, tumor markers, and urine analysis. X-axis represents z-score-normalized values. The median value of each variable for each group was annotated as a diamond-shaped figure on the bar.

**
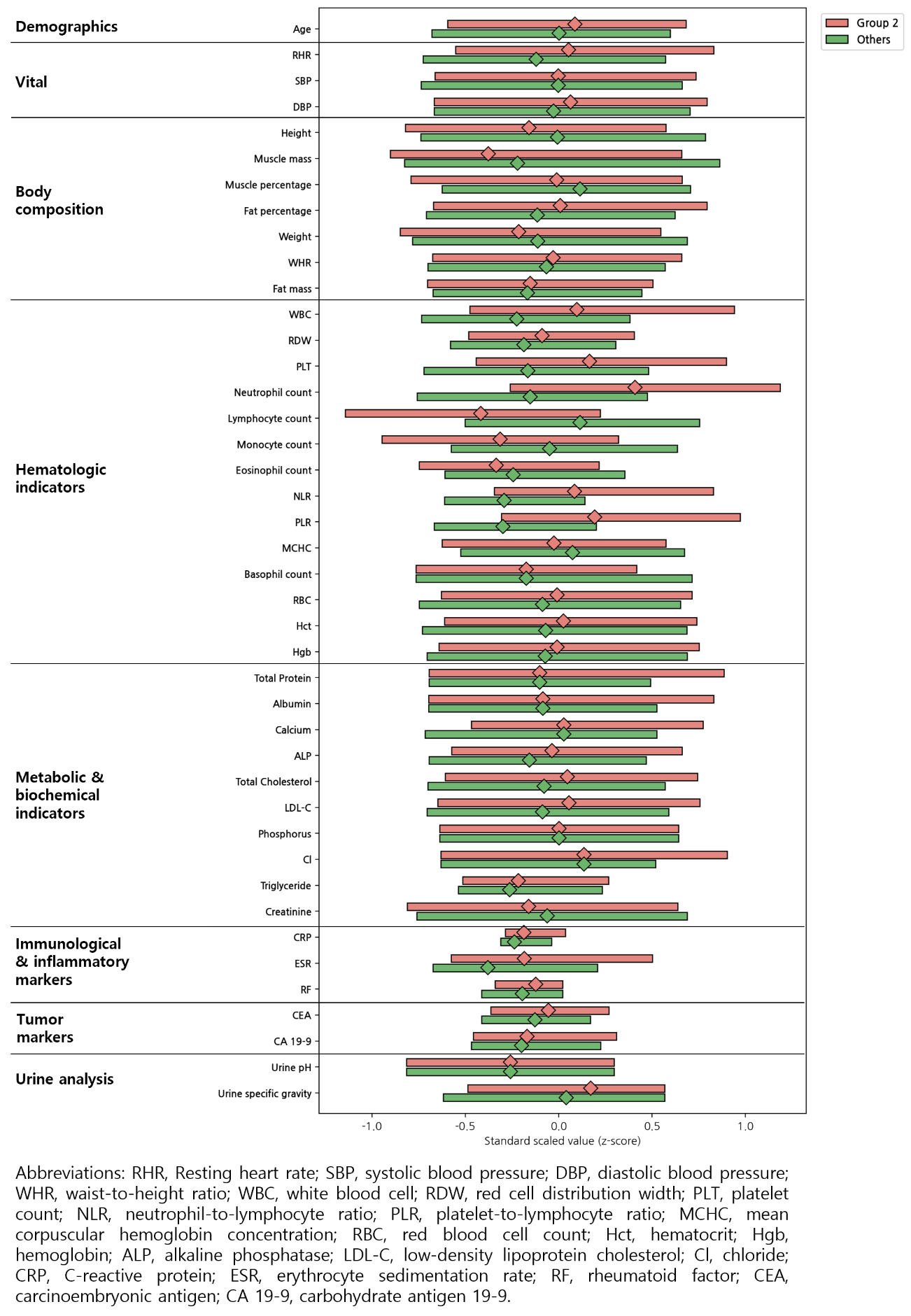
Supplementary Figure 5.** IQR plots of the statistically significant numerical variables according to binary NKA status (threshold: 500). Variables were grouped into eight categories which are demographics, vital, body composition, hematologic indicators, metabolic & biochemical indicators, immunological & inflammatory markers, tumor markers, and urine analysis. X-axis represents z-score-normalized values. The median value of each variable for each group was annotated as a diamond-shaped figure on the bar.


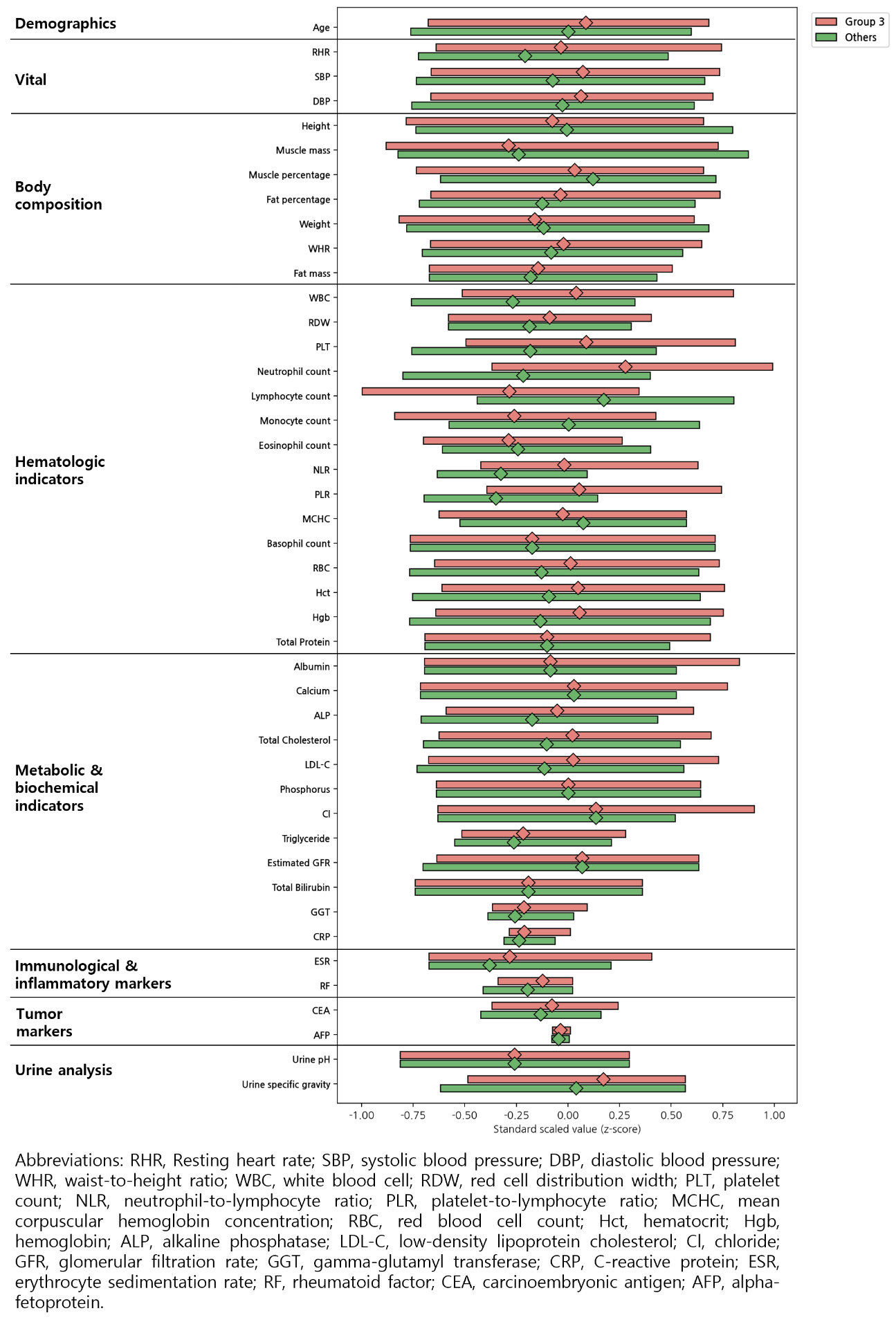


**Supplementary Figure 6.** IQR plots of NKA values for the statistically significant questionnaire items (with binary options). Variables were grouped into eight categories which are medical history, lifestyle, and current symptom. X-axis represents raw NKA values. The median value of each variable for each group was annotated as a diamond-shaped figure on the bar.


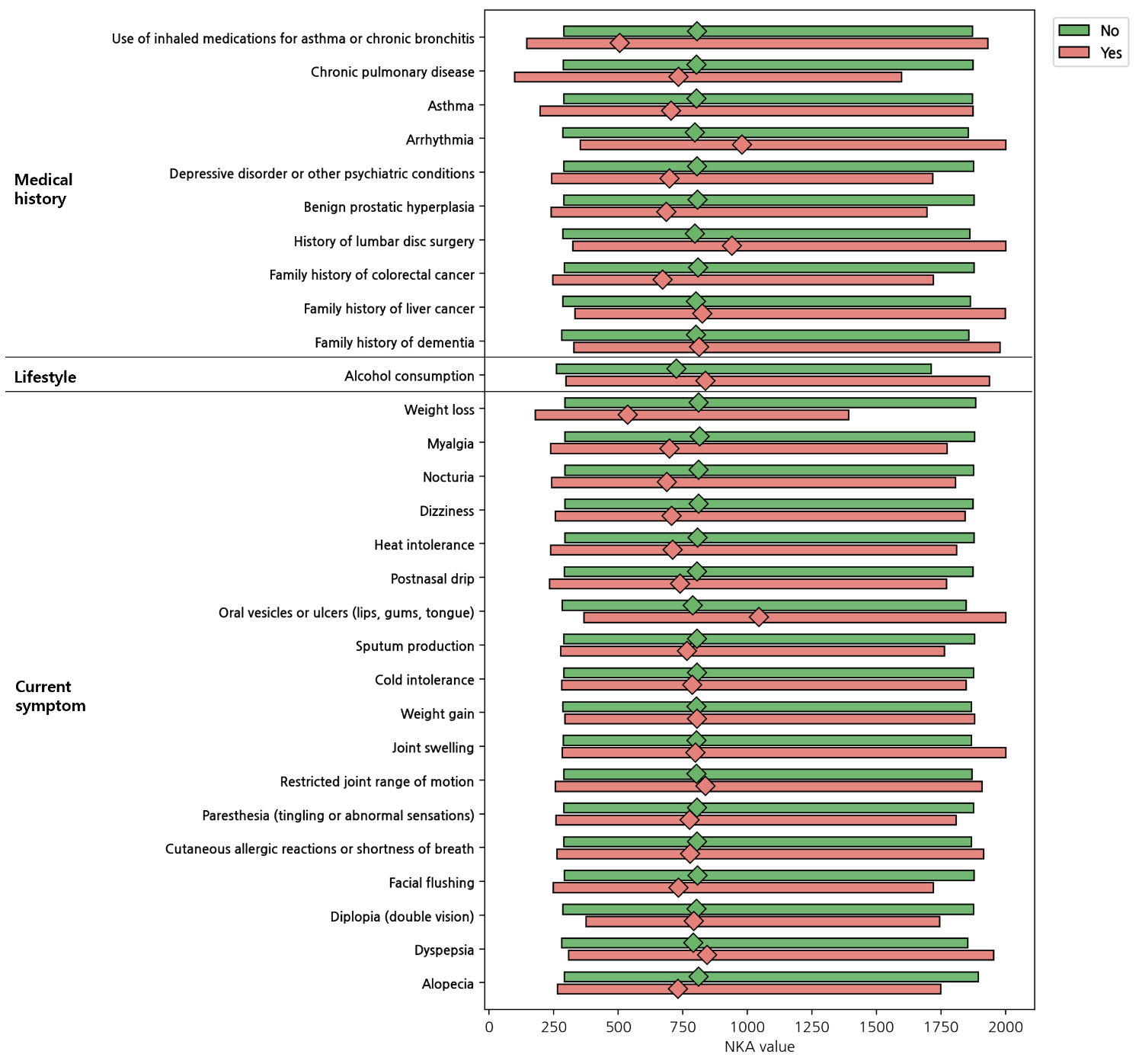


**Supplementary Figure 7.** IQR plots of NKA values for the statistically significant questionnaire items (with more than two options). X-axis represents raw NKA values. The median value of each variable for each group was annotated as a diamond-shaped figure on the bar.


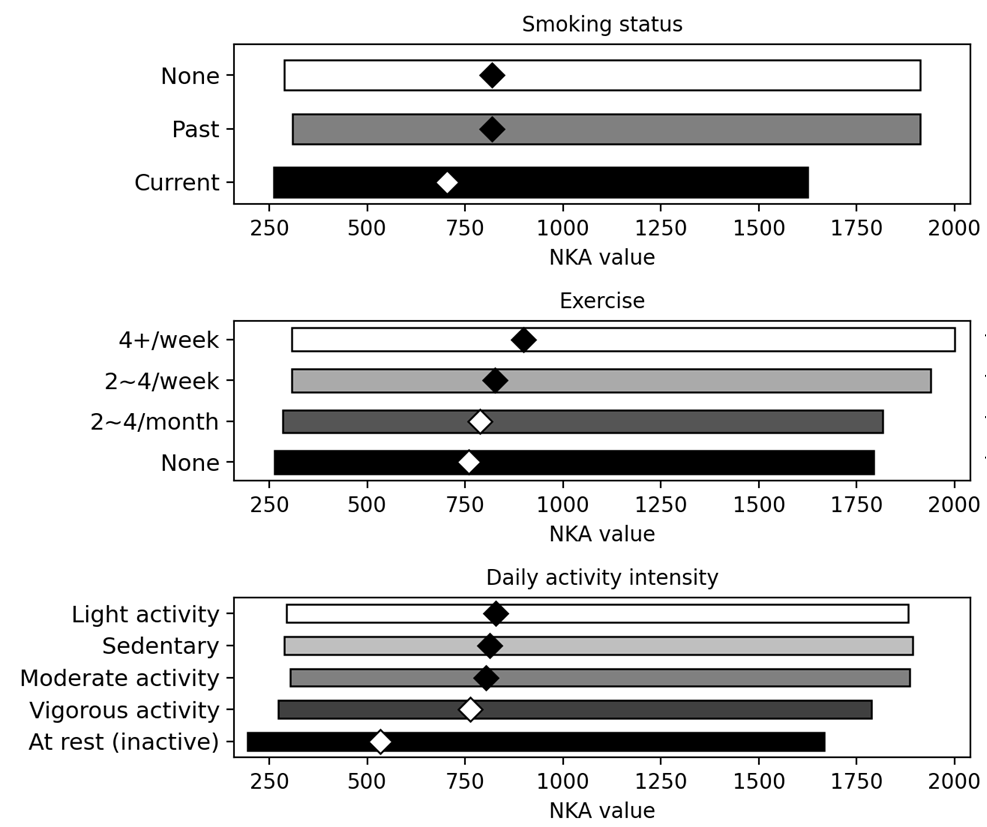

Supplement: Supplementary file 1 [file DataSheet1.docx]
